# In vivo screen of *Plasmodium* targets for mosquito-based malaria control

**DOI:** 10.1038/s41586-025-09039-2

**Published:** 2025-05-21

**Authors:** Alexandra S. Probst, Douglas G. Paton, Federico Appetecchia, Selina Bopp, Kelsey L. Adams, Tasneem A. Rinvee, Sovitj Pou, Rolf Winter, Esrah W. Du, Sabrina Yahiya, Charles Vidoudez, Naresh Singh, Janneth Rodrigues, Pablo Castañeda-Casado, Chiara Tammaro, Daisy Chen, Karla P. Godinez-Macias, Jasmine L. Jaramillo, Giovanna Poce, Michael J. Rubal, Aaron Nilsen, Elizabeth A. Winzeler, Jake Baum, Jeremy N. Burrows, Michael K. Riscoe, Dyann F. Wirth, Flaminia Catteruccia

**Affiliations:** 1 Department of Immunology and Infectious Diseases, Harvard T. H. Chan School of Public Health, Boston, MA USA; 2 VA Medical Center, Portland, OR USA; 3 Department of Life Sciences, Imperial College London, London, UK; 4 Harvard Center for Mass Spectrometry, Cambridge, MA USA; 5 Global Health Medicines R&D, GlaxoSmithKline, Madrid, Spain; 6 Department of Chemistry and Pharmaceutical Technologies, Sapienza University of Rome, Rome, Italy; 7 Department of Pediatrics, University of California, San Diego, La Jolla, CA USA; 8 Southwest Research Institute, San Antonio, TX USA; 9 Department of Molecular Microbiology and Immunology, Oregon Health and Science University, Portland, OR USA; 10 School of Biomedical Sciences, University of New South Wales, Sydney, New South Wales Australia; 11 Medicines for Malaria Venture, Geneva, Switzerland; 12 Infectious Disease and Microbiome Program, The Broad Institute, Cambridge, MA USA; 13 Howard Hughes Medical Institute, Boston, MA USA; 14 Present Address: Department of Infectious Disease, University of Georgia, Athens, GA USA

**Keywords:** Parasite biology, Phenotypic screening

## Abstract

The decline in malaria deaths has recently stalled owing to several factors, including the widespread resistance of *Anopheles* vectors to the insecticides used in long-lasting insecticide-treated nets (LLINs)^1,2^. One way to mitigate insecticide resistance is to directly kill parasites during their mosquito-stage of development by incorporating antiparasitic compounds into LLINs. This strategy can prevent onward parasite transmission even when insecticides lose efficacy^3,4^. Here, we performed an in vivo screen of compounds against the mosquito stages of *Plasmodium falciparum* development. Of the 81 compounds tested, which spanned 28 distinct modes of action, 22 were active against early parasite stages in the mosquito midgut lumen, which in turn prevented establishment of infection. Medicinal chemistry was then used to improve antiparasitic activity of the top hits from the screen. We generated several endochin-like quinolones (ELQs) that inhibited the *P.* *falciparum* cytochrome *bc*_1_ complex (CytB). Two lead compounds that targeted separate sites in CytB (Q_o_ and Q_i_) showed potent, long-lasting and stable activity when incorporated and/or extruded into bed net-like polyethylene films. ELQ activity was fully preserved in insecticide-resistant mosquitoes, and parasites resistant to these compounds had impaired development at the mosquito stage. These data demonstrate the promise of incorporating ELQ compounds into LLINs to counteract insecticide resistance and to reduce malaria transmission.

## Abstract (web summary)

An in vivo screen of small-molecule compounds to inhibit the mosquito-stage development of *Plasmodium* identified hits that can be incorporated into bed nets and led to effective parasite killing in the insect host.

## Main

After a consistent decline in cases and deaths at the start of this century, the burden of malaria has plateaued in recent years and in 2023, there were an estimated 263 million cases and 597,000 deaths^1^. Vector control of the anopheline mosquitoes that transmit malaria played a major part in this decline in cases, particularly the use of LLINs. Indeed, it was estimated that the use of LLINs accounted for up to 68% in the reduction of malaria prevalence in sub-Saharan Africa from 2000 to 2015 (ref. ^5^). However, widespread insecticide resistance jeopardizes the continued efficacy of these mainstay tools of vector control^1,2,6,7^, and different approaches that capitalize on the benefits of vector-targeted prevention while circumventing insecticide resistance are needed.

Interventions that directly target parasites in the mosquito represent a promising approach to disrupt parasite transmission and to reduce malaria burden^8–10^. We previously reported that the antimalarial drug atovaquone can be used in this way to directly kill *P.* *falciparum* in their anopheline vector^3,4^. Mosquitoes that landed on an atovaquone-coated surface before taking an infectious blood meal—in a manner analogous to how mosquitoes contact insecticides on LLINs or other surfaces in real life—exhibited inhibited development of parasites. Epidemiological modelling showed that the incorporation of a similarly potent antimalarial into existing LLINs would cause a significant reduction in malaria prevalence across sub-Saharan Africa, which was particularly prominent in regions of widespread insecticide resistance^3^.

Of note, the use of a *Plasmodium-*specific compound would not confer any fitness cost or selective pressure to the anopheline mosquito^3^, which therefore avoids potential development of resistance by the vector. Moreover, because the number of *Plasmodium* parasites during the mosquito stage of development is many orders of magnitude lower than during the asexual blood stages^11^, there is likely to be a lower propensity for de novo resistance-conferring mutations to emerge. The combination of compounds with different modes of action would further reduce the likelihood of resistance emerging in mosquito-stage parasites. In fact, the complementary use of different antiparasitic compounds in humans and in mosquitoes could act as a further barrier to the spread of resistance by disrupting the transmission of parasites resistant to frontline antimalarials. The identification of multiple compounds with antiplasmodial activity during mosquito stages is therefore a priority for the deployment of this strategy in the field.

## In vivo compound screen in mosquitoes

**Fig. 1:**
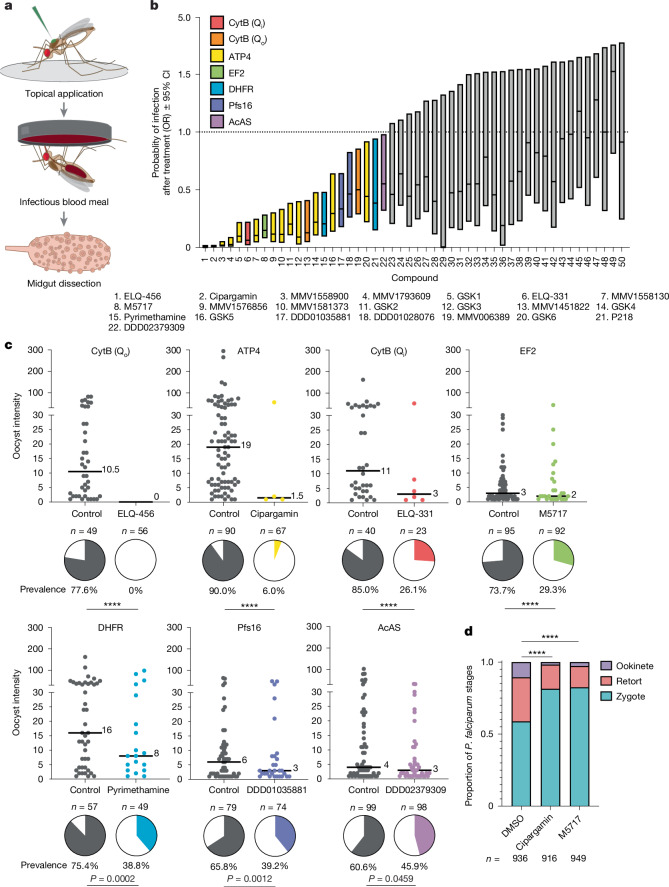
In vivo screen of 81 antiplasmodial compounds identifies seven *P.* *falciparum* targets essential during mosquito development. **a**, Schematic of the topical-application screen. A total of 2 mM of each antimalarial was solubilized in acetone and 2% DMSO and 0.5 µl was pipetted onto the dorsal thorax of female *A.* *gambiae* mosquitoes before being fed *P.* *falciparum-*infected blood. Midguts were dissected at 7 d.p.i. to detect oocysts. For compounds that reduced oocyst prevalence relative to the DMSO control, applications were repeated for two or more biological replicates. **b**, A total of 81 antiplasmodial compounds (spanning 28 modes of action) were screened (only the top 50 are shown here; see Supplementary Table 1 for the complete list of compounds and targets), and 22 compounds with 7 distinct targets significantly reduced *P.* *falciparum* prevalence. The odds ratio (OR) of compound-treated versus DMSO-control infection prevalence bounded by its 95% confidence interval (CI) is plotted (Baptista–Pike method). Compounds for which the upper confidence bound is below 1 significantly reduced *P.* *falciparum* prevalence and are shown in colour. See Supplementary Table 1 for complete OR and 95% CI values. **c**, The most active compounds in each hit target class (prevalence shown in pie charts, two-tailed Fisher’s exact test). ELQ-456 and ELQ-331, two biological replicates; cipargamin, pyrimethamine, DDD01035881 and DDD02379309, three biological replicates; M5717, four biological replicates. *n*, number of mosquitoes. Median lines and values are indicated. **d**, IFAs at 22 h.p.i. The ATP4 inhibitor cipargamin and the EF2 inhibitor M5717 disrupt parasite development during the zygote-to-ookinete transition. Ordinal logistic regression model, *n* = 3,640, d.f. = 19, *χ*2 (treatment) = 97.99, *P* = 4.20 × 10^−21^; *χ*2 (sample[treatment]) = 269.62, *P* = 4.80 × 10^−48^; DMSO versus cipargamin, d.f. = 1, *χ*2 = 29.8, *P* = 4.72 × 10^−8^; DMSO versus M5717, d.f. = 1, *χ*2 = 66.3, *P* = 3.33 × 10^−16^. Five biological replicates; *n*, number of total parasites counted. ****P ≤ 0.0001. Schematic in **a** adapted from ref. ^4^ under a Creative Commons licence CC BY 4.0. Source Data

To identify additional inhibitors of mosquito-stage *P.* *falciparum* development, we assembled a diverse library of small-molecule compounds with known asexual blood-stage antimalarial activity (Supplementary Table 1) and performed an in vivo topical-application screen (Fig. 1a). This screen was performed in vivo because despite recent advances^12^, there are no reliable methods to culture mosquito stages of *P.* *falciparum* development in vitro. In this topical system, compounds were solubilized in acetone and 2% dimethyl sulfoxide (DMSO) and directly pipetted onto the dorsal thorax of female *Anopheles gambiae* mosquitoes before *P.* *falciparum* infection. The 2% DMSO–acetone vehicle helped to disrupt the waxy mosquito cuticle and enhance compound membrane permeability, which enabled the partial bypass of potential barriers to facilitate uptake of the compounds. Mosquitoes were then provided with a blood meal that contained *P.* *falciparum*. At 7 days post-infection (d.p.i.), parasite burden was determined by counting oocysts present in the midgut.

Owing to the low-throughput nature of this in vivo assay, we limited our library to 81 antiplasmodial compounds, which comprised 28 distinct mechanisms of action that we hypothesized may be essential during the mosquito stage of *P.* *falciparum* development (Supplementary Table 1). We prioritized compounds with diverse targets that may be in the antimalarial developmental pipeline but are not currently used for frontline antimalarial treatment. We also considered multiple factors when selecting compounds, including antimalarial potency in blood stages and demonstration of multistage activity (for example, activity against liver schizonts or in standard membrane-feeding assays). This screen ultimately identified 22 compounds that significantly reduced parasite infection, and these compounds spanned seven *P.* *falciparum* targets (Fig. 1b). Targets included the ubiquinol oxidation (Q_o_) site of the *P.* *falciparum* cytochrome *bc*_1_ complex (CytB), which was expected because of results from our previous work with atovaquone^3^. The most effective Q_o_-site inhibitor was the endochin-like quinolone ELQ-456, which completely inhibited infection (that is, zero observed oocysts after treatment; Fig. 1c). Moreover, an inhibitor of the CytB ubiquinone reduction (Q_i_) site, ELQ-331 (ref. ^13^), strongly reduced infection prevalence (69.3% reduction in oocyst prevalence). This result provided further evidence for the necessity of CytB and mitochondrial activity for *P.* *falciparum* development in the mosquito. Other target hits included the sodium-proton antiporter P-type ATPase 4 (ATP4)^14,15^, with the clinical candidate cipargamin almost completely eliminating infection (93.3% prevalence reduction). The eukaryotic elongation factor 2 (EF2) was also an identified target hit, and the inhibitor M5717 (ref. ^16^) (previously known as DDD107498, proposed trade name cabamiquine) showed significant activity (60.2% prevalence reduction; Fig. 1c). The remaining most active hits from each target class included the following compounds: pyrimethamine (49.6% prevalence reduction), which targets dihydrofolate reductase (DHFR, the essential bifunctional enzyme in folate metabolism and nucleotide biosynthesis^17^); DDD01035881 (40.4% prevalence reduction), which targets the parasitophorous vacuole membrane protein S16 (Pfs16, an early surface marker of gametocytes with a role in microgametogenesis^18,19^); and DDD02379309 (20.8% prevalence reduction), which targets acetyl coenzyme A synthetase (AcAS, the enzyme essential for the acetylation of CoA and has downstream roles in the tricarboxylic acid cycle, lipid synthesis and histone acetylation^20,21^) (Fig. 1b,c).

We previously showed that the CytB inhibitor atovaquone disrupts parasite development in the midgut of mosquitoes during the zygote–ookinete transition^3^. To determine the specific stage of action of two of our most active compounds, the ATP4 inhibitor cipargamin and the EF2 inhibitor M5717, we performed immunofluorescence assays (IFAs) on mosquito midguts at 22 h post-infection (h.p.i.). Both compounds induced a significant decrease in the proportion of ookinetes relative to controls. This result suggests that both Na^+^ ion homeostasis and protein synthesis play a crucial part in the proper differentiation of parasites from the fertilized zygote to the elongated ookinete (Fig. 1d).

For most of the mechanisms of action in this screen, we saw variable activity across multiple compounds with the same target. For example, the alkoxy carbonate ester prodrugs ELQ-456 (CytB Q_o_-site inhibitor) and ELQ-331 (CytB Q_i_-site inhibitor)^13,22^ significantly reduced *P.* *falciparum* prevalence. However, their active forms (ELQ-437 and ELQ-300, respectively) did not display activity even though they had similar asexual blood-stage potency (Extended Data Table 1 and Supplementary Table 1), which could be due to differential uptake through the cuticle.

Notably, most of the mechanisms of action that were targeted (21 out of 28) could not be inhibited in our screen. For example, none of the seven inhibitors of dihydroorotate dehydrogenase (DHODH, the rate-limiting enzyme in parasite pyrimidine biosynthesis) were active, including the potent former clinical candidate DSM265 (ref. ^23^) (Supplementary Table 1). Rather than reflecting a nonessential target, this lack of activity may be due to poor compound uptake, degradation by mosquito metabolic enzymes or other elements of parasite or mosquito pharmacokinetics. Indeed, it is plausible that several compounds tested in our screen may simply possess unfavourable chemical characteristics for mosquito-stage inhibition of the parasite.

**Extended Data Fig. 1:**
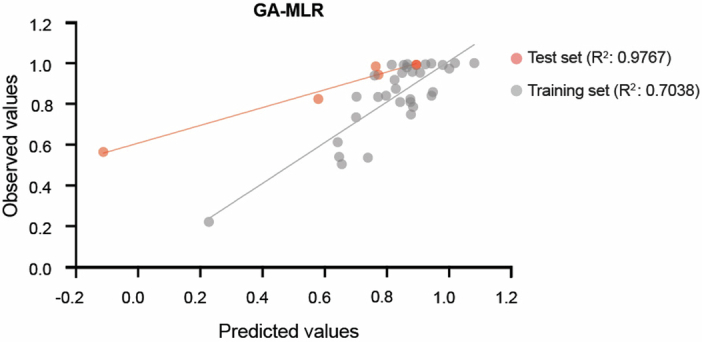
Correlation plot for GA-MLR QSAR model. Comparison of predicted (x-axis) and observed (y-axis) topical-application activity. Predicted values were generated with a genetic algorithm-multiple linear regression (GA-MLR) model, which produced a 0.70 R^2^ (coefficient of determination) between our observed and predicted topical-application activity. Gray dots represent compounds used in the model training set and orange dots represent compounds used in the model test set. Goodness-of-fitness for each set is represented by the R^2^ values. Source Data

As a first step to addressing this issue and to determine whether there were any shared physicochemical features among our hit compounds, we generated an in silico quantitative structure–activity relationship (QSAR) model using both active and inactive inhibitors of the seven essential targets identified here (Extended Data Fig. 1). With the caveat that our QSAR analysis could only include 38 compounds (25 positive and 13 negative), we found that lipophilicity (GCUT-SLOGP-3) and concentration of regions of molecular hydrophobicity (vsurf-ID8) were positive and negative predictors of compound activity, respectively (Supplementary Table 2). This result suggests that an even distribution of lipophilicity across a molecule is beneficial for topical-application activity. Negative compounds (that is, had no activity), for which we had no knowledge on whether their targets have a pivotal role in this portion of the parasite life cycle, could not be incorporated in the analysis. Future studies will be needed to conclusively determine whether these compounds are not suitable for mosquito-based inhibition or whether their targets (including DHODH) are not essential in the early stages of *P.* *falciparum* development in the mosquito.

## Mosquito tarsal-contact assays

**Fig. 2:**
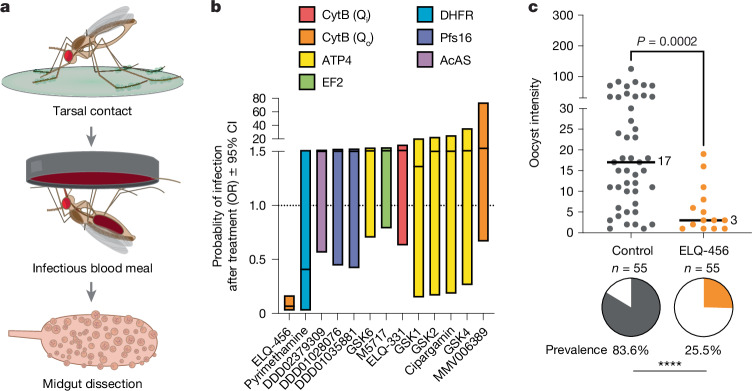
Most compounds are inactive in tarsal-contact assays. **a**, Schematic of the tarsal-contact experiment. Mosquitoes land on a surface coated with 1 mmol m^–2^ compound or vehicle control for up to 6 min. One hour later, exposed mosquitoes are provided with an infectious blood meal. At 7 d.p.i., mosquito midguts are dissected. **b**, Of the topically active compounds tested (colour coded on the basis of the target), only the endochin-like quinolone ELQ-456 demonstrated antiparasitic activity after tarsal uptake. The OR of compound-treated versus vehicle-control infection prevalence bounded by its 95% CI is plotted (Baptista–Pike method). An upper confidence bound below 1 signifies reduced *P.* *falciparum* prevalence. Tarsal-contact assays were repeated for 2 biological replicates for ELQ-456 (control, *n* = 55; treatment, *n* = 55) and pyrimethamine (control, *n* = 50; treatment, *n* = 42) and one replicate for the other compounds (DDD02379309 control, *n* = 41, treatment, *n* = 39; DDD01028076 control, *n* = 20, treatment, *n* = 25; DDD01035881 control, *n* = 20, treatment, *n* = 16; GSK6 control, *n* = 18, treatment, *n* = 26; M5717 control, *n* = 41, treatment, *n* = 38; ELQ-331 control, *n* = 24, treatment, *n* = 36; GSK1 control, *n* = 27, treatment, *n* = 17; GSK2 control, *n* = 27, treatment, *n* = 20; cipargamin control, *n* = 27, treatment, *n* = 22; GSK4 control, *n* = 27, treatment, *n* = 31; MMV006389 control, *n* = 24, treatment, *n* = 24). See Supplementary Table 1 for exact OR and 95% CI values**. c**, Tarsal contact with ELQ-456 causes a significant reduction in the prevalence (pie charts, two-tailed Fisher’s exact test) and intensity (two-tailed Mann–Whitney test) of infection. *n*, number of mosquitoes dissected. Median lines and values are indicated. ****P ≤ 0.0001. Schematic in **a** adapted from ref. ^4^ under a under a Creative Commons licence CC BY 4.0. Source Data

**Fig. 3:**
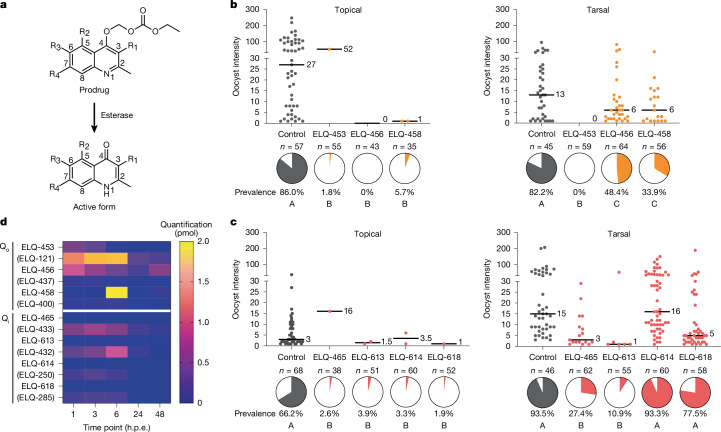
Modifications in ELQ structures enhance tarsal-mediated inhibition. **a**, ELQ core scaffold indicating all residues discussed in the text. The alkoxy carbonate ester promoiety at C4 is rapidly converted to its active form in vivo by parasite and/or host esterases^13^. **b**,**c**, Topical application of Q_o_-site (**b**) and Q_i_-site (**c**) inhibitors uniformly inhibited *P.* *falciparum* prevalence (pie charts, two-tailed Fisher’s exact test with post hoc Bonferroni multiple-test correction), whereas tarsal-contact activity depended on the chemical structure (shown for each compound in Extended Data Table 1). Two biological replicates were performed. *n*, number of mosquitoes dissected. Median lines and values are indicated. Letters in pie charts indicate significant differences in prevalence between groups (*P* < 0.05). The difference in ELQ-456 tarsal-contact activity between Fig. 2c and this figure is within standard variability for partially active compounds. **d**, LC–MS quantification of ELQ prodrugs and active forms (indicated in parentheses) in mosquito midguts at different time points after tarsal-contact exposure. Each time point represents the average of two biological replicates with two technical replicates each consisting of five pooled midguts. Source Data

In translational applications, a mosquito-targeted antimalarial would need to be taken up by the mosquito tarsi (legs) after landing on a drug-treated surface, such as a bed net. Thus, we tested the activity of at least one hit compound from each identified target class in tarsal-contact assays. In these experiments, mosquitoes were allowed to briefly (up to 6 min) land on a glass surface coated with the compound of interest before infection^3,4^ (Fig. 2a). In this assay, a much smaller mosquito surface area is exposed to the compound (only their tarsi rather than most of the thorax cuticle), and compound permeability is not assisted by the use of the DMSO–acetone vehicle. Of the 13 compounds we tested in tarsal-contact assays, only our most potent hit in the topical screen, ELQ-456 (CytB Q_o_-site inhibitor) reduced infection (69.5% reduction in oocyst prevalence) (Fig. 2b,c). All other compounds, including our second most active hit in the topical assays cipargamin and four additional ATP4 inhibitors, did not demonstrate activity. ELQ-331 (CytB Q_i_-site inhibitor) was also inactive in tarsal-contact assays despite sharing a core structural scaffold with ELQ-456 (core ELQ structure shown in Fig. 3a, substituents shown in Extended Data Table 1). Owing to the limited number of compounds tested, we were unable to perform a similar QSAR analysis for these compounds, but the data further indicate that even small differences in structure and physicochemical properties can have a substantial effect on tarsal uptake and activity.

## Enhancing ELQ activity in tarsi

On the basis of these results, we next asked whether changing various aspects of chemical structure could improve tarsal-based efficacy of the antimalarials tested. To this aim, we focused on ELQs given that ELQ-456 was our primary hit that showed efficacy in the tarsal-contact assays. Moreover, ELQs are attractive candidates because of their relatively scalable three-step synthesis (Methods) and promising cost-effectiveness properties. We applied a medicinal chemistry approach to generate additional Q_o_-site-targeting ELQ prodrugs (ELQ-453 and ELQ-458) with various 3-position moieties (Fig. 3a and Extended Data Table 1). ELQ-456 has an 11-carbon alkyl chain in this position whereas ELQ-453 has a 7-carbon alkyl chain and ELQ-458 has a diaryl ether group. These compounds showed strong and comparable activity against asexual blood-stage parasites in vitro (Extended Data Table 1) and in topical-application assays in mosquitoes (Fig. 3b). However, we observed marked differences in tarsal-contact assays. Specifically, ELQ-458 showed partial activity, similar to ELQ-456 (58.8% prevalence reduction), whereas ELQ-453 completely prevented *P.* *falciparum* infection (Fig. 3b). We also performed topical assays with the active forms of these compounds, which do not have the alkoxy carbonate ester promoiety. Similar to the active form of ELQ-456 (ELQ-437), the active form of ELQ-458 (ELQ-400) did not exhibit activity, whereas we detected modest activity with the active form of ELQ-453 (ELQ-121) (Extended Data Table 1). The observed poor activity of the active forms of ELQs may be explained by their propensity for poor aqueous solubility and high crystallinity, negative properties that are mitigated by prodrug formulations^13^.

**Extended Data Fig. 2:**
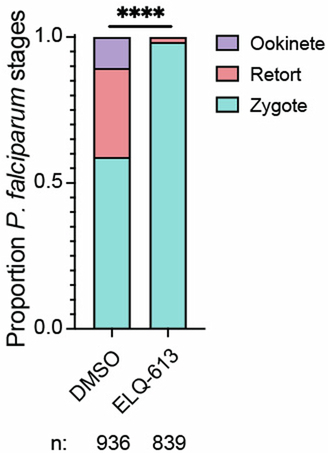
ELQ-613 stage specificity. Immunofluorescence assays performed at 22 h post infection demonstrated that the Cyt B Q_o_-site inhibitor ELQ-613 disrupts parasite development during the zygote to ookinete transition. Five biological replicates, n = number of total parasites counted. (Ordinal Logistic Regression model, n = 3640, df = 19, χ2 (Treatment) = 97.99, p =; χ2 (Sample[Treatment]) = 269.61, p =; DMSO-ELQ613 comparison: df = 1, χ2 = 25.5, p = 4.50 × 10^−7^). **** p-value ≤ 0.0001. Source Data

We next designed ELQ Q_i_-site prodrugs with modifications to their 3-position side chains (Fig. 3a and Extended Data Table 1) and tested them for improvements in tarsal-based uptake efficacy relative to the inactive ELQ prodrug ELQ-331. We synthesized compounds with a seven-carbon alkyl chain (ELQ-613), analogous to the most active Q_o_-site inhibitor ELQ-453, and a shorter, four-carbon alkyl chain (ELQ-618). We also varied the halogen group at the 6-position from a chlorine to a fluorine (seven-carbon alkyl chain, ELQ-465; four-carbon alkyl chain, ELQ-614). These four compounds potently inhibited asexual blood-stages (Extended Data Table 1) and were strongly active against mosquito stages in topical-application assays (Fig. 3c). In the latter, parasite killing most probably occurred during the zygote–ookinete transition, as indicated by IFAs with ELQ-613 (Extended Data Fig. 2), similar to our previous observations with atovaquone^3^. Notably, the length of the alkyl side chain made a significant difference when we tested the compounds in tarsal-contact assays. ELQ-613 and ELQ-465 (seven-carbon chain) significantly outperformed ELQ-614 and ELQ-618 (four-carbon chain) and severely inhibited infection (88.3% and 70.7% prevalence reduction, respectively) (Fig. 3c). The change in halogen group also affected efficacy, but to a lesser degree. That is, the 6-position chlorine compounds caused a greater reduction in prevalence of infection than the 6-fluorine analogues with the same 3-alkyl side-chain length (pie charts in Fig. 3c).

We next aimed to determine whether the degree of uptake in the tarsal-contact assays correlated with activity of the seven Q_o_-site and Q_i_-site ELQ prodrugs developed. To that end, we performed liquid chromatography and mass spectrometry (LC–MS) to measure levels of the prodrugs and their respective active forms in midgut tissues of mosquitoes after tarsal-contact assays. ELQ prodrugs have little to no activity against isolated *P.* *falciparum* mitochondria^13^ and need to be enzymatically converted to their active forms, probably by host esterases. ELQ-121, the active form of the most effective Q_o_-site inhibitor ELQ-453, showed the most abundant levels in LC–MS analyses, with a maximum of 1.69 pmol of compound per five pooled midgut tissues (0.34 pmol per midgut) at 6 h post exposure (h.p.e.) (Fig. 3d). Among the Q_i_-site inhibitors, ELQ-432 and ELQ-433, active forms of ELQ-613 and ELQ-465, respectively, had the highest levels measured at all time points (maximum of 0.9 and 0.7 pmol per five pooled midgut tissues at 6 and 3 h.p.e., respectively). By contrast, the largely inactive ELQ-614 and ELQ-618 active forms had low levels measured at all time points (maximum of 0.2 pmol per five pooled midgut tissues at 3 h.p.e. for both compounds) (Fig. 3d). These results highlight that uptake through mosquito tarsi is strongly affected by the structure of the compound and is key to its activity against *P.* *falciparum*.

## Combination of Q_o_–Q_i_-site ELQ inhibitors

**Fig. 4:**
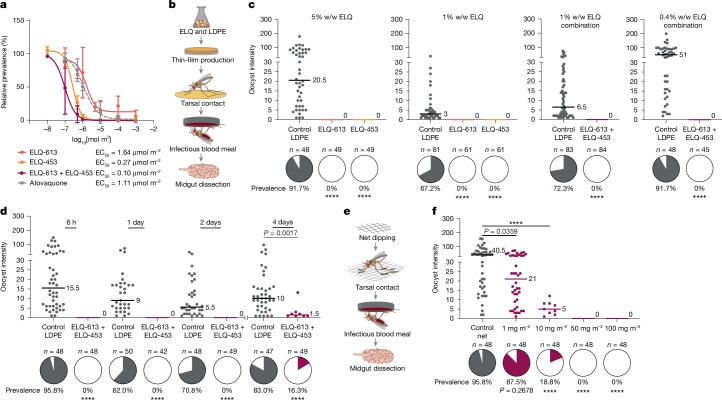
ELQ combination improves potency and is highly active in bed net-like materials. **a**, Tarsal contact dose–response curves for ELQ-453 (*n* = 6 biological replicates) and ELQ-613 (*n* = 4 biological replicates) alone or in combination (*n* = 3 biological replicates) and atovaquone (*n* = 2 biological replicates) (1 mmol m^–2^ to 0.01 µmol m^–2^). Each point represents the mean infection prevalence ± s.d. relative to a vehicle control. EC_50_ values were calculated from interpolated nonlinear regression dose–response curves. **b**, Schematic of mosquito exposure to LDPE thin films impregnated with ELQ compounds. **c**, ELQ-613 and ELQ-453 alone at 5% w/w and 1% w/w in LDPE, and in combination at 1% w/w (0.5% each) and 0.4% (0.2% each), completely inhibited parasite infection. **d**, Time course of tarsal-contact exposure to 1% w/w ELQ combination LDPE film showing complete parasite elimination when performed at 2 days and considerable reduction at 4 days pre-infection (the latter performed in a separate experiment). **e**, Dipped polyester net tarsal-contact schematic (Methods). **f**, Tarsal contact with nets dipped with ELQ-613 and ELQ-453 was completely effective at 50 mg m^–2^ (25 mg m^–2^ each ELQ), with some activity detected at as low as 1 mg m^–2^. In **c**, **d** and **f**, two biological replicates were performed. *n*, number of mosquitoes. Median lines and values are indicated. Prevalence (pie charts) significance calculated using two-tailed Fisher’s exact test with post hoc Bonferroni multiple-test correction. Intensity significance was calculated using two-tailed Mann–Whitney (**d**) or Kruskal–Wallis test with Dunn’s post hoc multiple comparisons correction (**f**). ****P ≤ 0.0001. Schematics in **b** and **e** adapted from ref. ^4^ under a under a Creative Commons licence CC BY 4.0. Source Data

Combination therapy is now considered the mainstay for malaria therapeutics because it improves efficacy and reduces the probability of rapid emergence of parasite resistance compared with monotherapy^24^. We therefore used tarsal-contact assays to test whether CytB Q_o_-site and Q_i_-site (Q_o_–Q_i_-site) inhibitors could be used in combination to block the mosquito transmission of parasites. In dose–response curves, when used in a 1:1 ratio, the ELQ-453 and ELQ-613 combination was more potent at inhibiting *P.* *falciparum* infection than either compound alone as well as atovaquone, with an interpolated half maximal-effective concentration (EC_50_) of 0.10 µmol m^–2^ (Fig. 4a).

**Extended Data Fig. 3:**
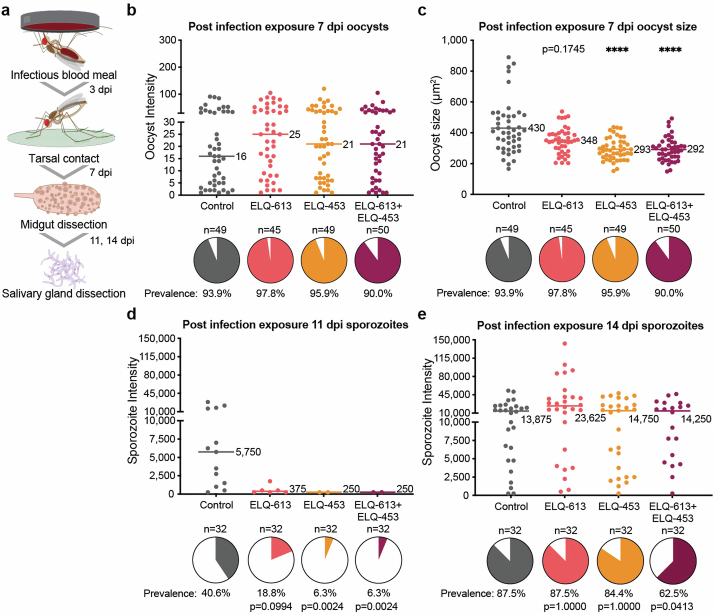
Post infection ELQ exposure. **a**, Tarsal contact schematic. Assays were performed as previously described but at 3 days post-infection rather than prior to an infectious blood meal. **b**, Post-infection tarsal contact with ELQ-453 and ELQ-613 alone or in combination did not significantly alter the prevalence or intensity of parasites, but **c**, caused a significant reduction in oocyst size in the ELQ-453 and combination groups. Oocyst cross-sectional area significance calculated via Kruskal-Wallis test with Dunn’s post hoc multiple comparisons correction. **d**, We detected significantly lower prevalence of sporozoite infection (6.3%) in the same two groups at 11 dpi, an early time point when instead 40% of control females were carrying sporozoites. **e**, At 14 dpi, the prevalence of sporozoites in the combination exposed group had a 28.6% reduction in sporozoite prevalence relative to controls. For **b**, **d**, and **e**, median lines and values indicated, and for **c**, average line and values indicated. For **b-e**, n = number of mosquitoes dissected, two biological replicates were performed, prevalence significance (pie charts) was calculated via two-tailed Fisher’s exact test. **** indicates p-value ≤ 0.0001. Schematic in **a** adapted from ref. ^4^ under a Creative Commons licence CC BY 4.0. Source Data

We next tested whether these compounds could interfere with ongoing infections by performing tarsal-contact assays with ELQ-453 and ELQ-613 either alone or in combination at 3 d.p.i., when oocysts are established in the midgut and beginning to grow (Extended Data Fig. 3a). In female mosquitoes that were briefly exposed (up to 6 min) to ELQ-453 alone or in combination with ELQ-613, oocyst size (a direct proxy for parasite developmental rates^25^) was decreased at 7 d.p.i., a midpoint in oocyst development (31.8% reduction with ELQ-453 alone compared with 32.1% reduction with the combination relative to controls). This decrease was also reflected at the sporozoite level at an early time point after infection (11 d.p.i.), when a much lower number of female mosquitoes were infected with salivary-gland sporozoites (6.3% prevalence of infection) in the same two treatment groups relative to controls (40% prevalence of infection) (Extended Data Fig. 3b–d). Despite similar results, the effect of ELQ-613 alone was not significant. At 14 d.p.i., a time point at which most control female mosquitoes (87.5%) had sporozoites in their salivary glands, the ELQ combination led to reduced sporozoite prevalence relative to controls (28.6% reduction in prevalence; Extended Data Fig. 3e). These data demonstrate that a single, short period of mosquito contact with surfaces treated with the ELQ combination can both prevent establishment of infection and slow down ongoing infections, thereby extending the parasite developmental time in the mosquito (the extrinsic incubation period (EIP)). These results are promising, as extending the EIP would further increase the impact of these compounds on malaria transmission dynamics^26^ given the relatively short lifespan of mosquitoes in the field^27–29^.

## Activity in bed net-like substrates

**Extended Data Fig. 4:**
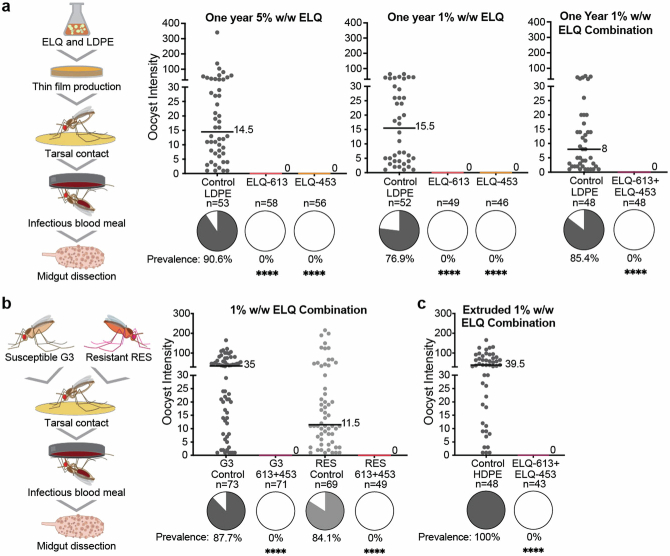
Polymer film longevity, activity against insecticide resistant mosquitoes, and extrusion testing. **a**, Low density polyethylene (LDPE) ELQ thin films were generated as described in Fig. 4. 5%, 1%, and 1% combination w/w ELQ-613 and ELQ-453 films retained complete inhibitory activity over one year after they were first generated after being stored at ambient room temperature (~21 °C) with regular day-night light exposure. **b**, Tarsal-contact exposures were performed using insecticide resistant *An. gambiae* (RES line)^31^. Contact with 1% w/w combination ELQ films completely inhibited infection in both insecticide resistant (RES) and susceptible (G3) *An. gambiae*. **c**, Tarsal contact with high density polyethylene films incorporated with 1% w/w ELQ (0.5% ELQ-453, 0.5% ELQ-613), extruded into a polymer, and flattened into a film completely inhibited parasite infection. In **a** and **c**, two biological replicates were performed and in **b**, three biological replicates were performed. n = number of mosquitoes dissected, median lines and values indicated, prevalence significance was calculated via two-tailed Fisher’s exact test with post hoc Bonferroni multiple test correction in **b** and **c**. **** indicates p-value ≤ 0.0001. Schematics in **a** and **b** adapted from ref. ^4^ under a Creative Commons licence CC BY 4.0. Source Data

For use in LLINs, compounds need to retain their activity in a three-dimensional polymer and under the high heat conditions necessary for their incorporation and extrusion in such textiles. To test whether ELQ-453 and ELQ-613 would maintain efficacy in a bed net-like formulation, we generated low-density polyethylene (LDPE) three-dimensional polymer films at high heat (150 °C) using the two compounds either alone or in combination at 5%, 1% and 0.4% by weight (w/w; Fig. 4b). These concentrations are broadly in line with the concentration of insecticides used in polyethylene LLINs, which vary from 0.1% to 10% w/w depending on the active ingredient^30^. Brief tarsal-contact exposure to ELQ-impregnated LDPE films completely ablated *P.* *falciparum* infection, as determined by the absence of oocysts in all treatment groups relative to a polyethylene control film with no compounds incorporated (Fig. 4c). Crucially, these films maintained their antiplasmodial activity when tested 1 year later after being stored at room temperature with light exposure and when tested in an insecticide-resistant *A.* *gambiae* strain^31^ (Extended Data Fig. 4a,b). These results confirm the stability of these compounds after their incorporation in polymers and their activity in field-relevant mosquitoes. When testing the duration of protection, parasite infection was fully prevented when female mosquitoes were exposed to a film with 1% ELQ-453 and ELQ-613 2 days before infection. Even at 4 days after exposure, we observed a substantial reduction in prevalence and intensity of infection (Fig. 4d). These results demonstrate that the prophylactic value of a net would extend beyond the time of contact, and that mosquitoes that are exposed to the compounds one evening but bite an infected person over subsequent days would not support parasite development.

To test ELQ activity in another common net formulation, we performed polyester net-dipping experiments (Fig. 4e). Polyester nets were dipped in a 1:1 combination of ELQ-453 and ELQ-613 in acetone. These nets were allowed to dry, and subsequent tarsal-contact experiments showed potent, dose-dependent activity, with complete inhibition of parasite infection at concentrations as low as 50 mg m^–2^ (Fig. 4f). Finally, the ELQ combination retained full activity when directly extruded in high-density polyethylene (HDPE) ELQ films at 1% w/w (0.5% w/w each compound) using a small-scale extrusion machine (Extended Data Fig. 4c). This process mimics the procedure for generating compound-incorporated yarn that is then knit into bed nets. Altogether, these data provide strong support for the potential use of these inhibitors for field applications.

## No Q_o_–Q_i_-site mutant cross-resistance

**Fig. 5:**
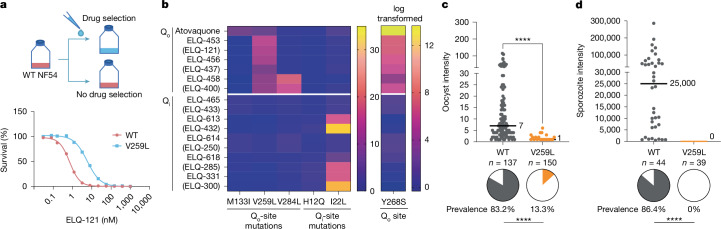
Q_o_-site mutants are susceptible to Q_i_-site inhibitors and have impaired sporogony in the mosquito. **a**, Schematic of drug selection of ELQ-121 (active form of ELQ-453). **b**, Fold-change asexual blood-stage EC_50_ values for CytB mutants relative to control wild-type (WT) NF54. Q_o_-site mutants retained susceptibility to Q_i_-site inhibitors, and vice versa, in asexual blood stages. The clinical isolate TM90-C2B has a >13,000-fold EC_50_ shift to atovaquone, and therefore was log-transformed and plotted separately for clarity. **c**,**d**, The Q_o_-site-resistant V259L parasites produced few oocysts (**c**; five biological replicates) and had no sporozoites (**d**; three biological replicates). *n*, number of mosquitoes dissected. Median lines and values are indicated. Prevalence significance was calculated using two-tailed Fisher’s exact tests and intensity was calculated using two-tailed Mann–Whitney tests. ****P ≤ 0.0001. Source Data

In addition to the benefit of using a combination of Q_o_–Q_i_-site inhibitors, such a dual-targeting strategy would slow the emergence of parasite drug resistance during mosquito stages. We therefore assessed the propensity for cross-resistance between Q_o_-site and Q_i_-site inhibitors by performing drug selections in transmissible NF54 parasites with the Q_o_-site inhibitors ELQ-121 (the active form of ELQ-453) and ELQ-400 (the active form of ELQ-458) and the Q_i_-site inhibitors ELQ-432 (the active form of ELQ-613) and ELQ-300 (the active form of ELQ-331), and with atovaquone (Fig. 5a). From these selections, we generated a panel of resistant parasite lines with nonsynonymous point mutations (Q_o_-site, M133I, V259L and V284L; Q_i_-site, H12Q) (Extended Data Table 2). The lone Q_i_ mutant emerged from multiple selections with ELQ-300, whereas multiple attempts at selection with ELQ-432 failed to produce resistant parasites, a result consistent with previous attempts showing that Q_i_-site inhibitors have a low propensity for in vitro resistance^32,33^. To this panel of resistant mutants, we added D1, a previously selected Dd2 clone with an I22L Q_i_-site mutation^33^, and TM90-C2B, an atovaquone-resistant clinical isolate with the Y268S Q_o_-site mutation^34^.

In asexual blood-stages, some Q_o_-site mutations were highly compound-specific, such as the V284L mutation (selected with ELQ-400), which only conferred resistance to ELQ-400 and its prodrug ELQ-458 (EC_50_ shift of about 15-fold). Other mutations such as V259L conferred some level of resistance (EC_50_ shift of about 5–20-fold) to all of the tested Q_o_-site inhibitors (Fig. 5b), whereas the M133I mutation conferred only low-level atovaquone resistance (EC_50_ shift of about 5-fold) and seemed to sensitize parasites to Q_o_-site ELQ inhibitors. Among the Q_i_-site mutants, the D1 clone demonstrated moderate resistance (EC_50_ shift of about 2–30-fold) to our panel of Q_i_-site inhibitors, whereas the H12Q mutant had <2-fold resistance to all the tested Q_i_-site compounds. Notably, none of the parasite lines with mutations in their Q_o_-site were resistant to the tested Q_i_-site inhibitors and vice versa (Fig. 5b).

## ELQ mutants have impaired sporogony

**Extended Data Fig. 5:**
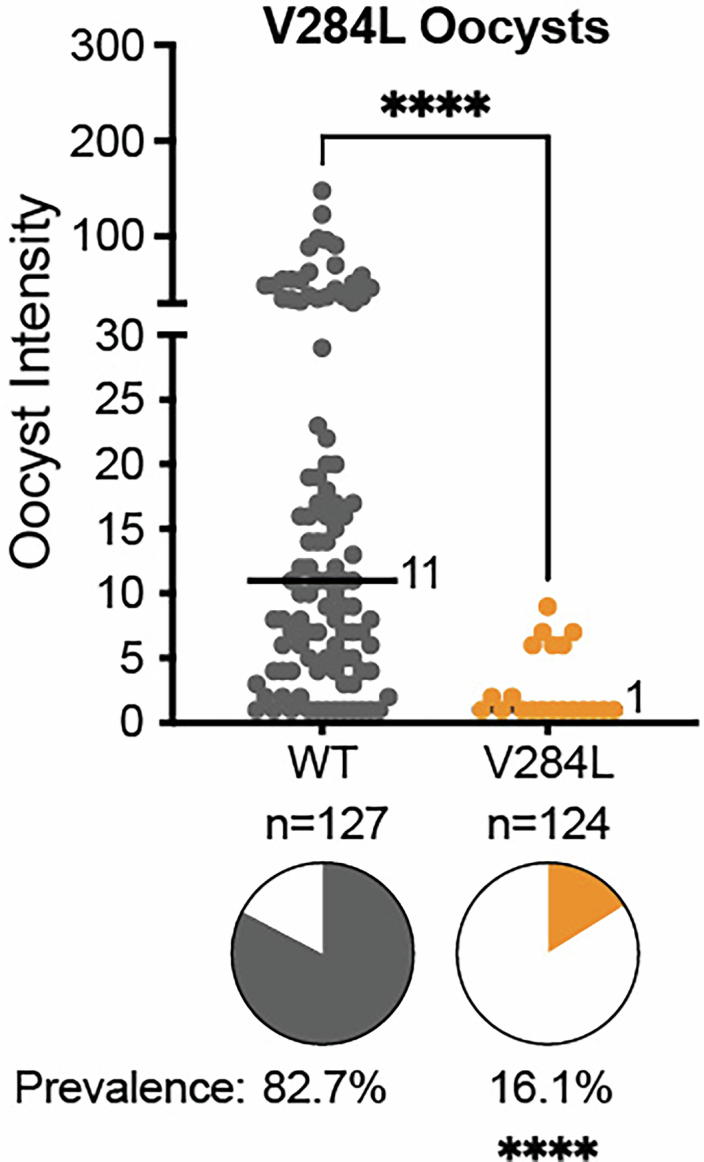
V284L mutants show low oocyst intensity and prevalence of infection. Parasites with the Cyt B Q_o_-site mutation V284L have a significant decline in oocyst intensity and prevalence relative to controls at 7 dpi. Four biological replicates, median lines and values indicated, n = number of mosquitoes dissected, prevalence significance calculated via two-tailed Fisher’s exact test and intensity calculated via two-tailed Mann-Whitney. **** indicates p-value ≤0.0001. Source Data

Severe defects in the sporogony and transmission of CytB mutants have been previously reported^35–37^. We confirmed these findings using our V259L Q_o_-site mutant, which showed some level of resistance (EC_50_ shift of about 5–20-fold) to all Q_o_-site inhibitors in our panel (Fig. 5b). After infection with the V259L mutant, mosquitoes showed a marked decrease in oocyst prevalence (13.3% compared with 80% in controls) and intensity (a median of 1 and maximum of 6 oocysts compared with a median of 7 and maximum of 115 oocysts in controls) (Fig. 5c). Moreover, when we dissected salivary glands at 14 d.p.i., no sporozoites were observed in mosquitoes infected with the V259L mutant, whereas controls had high sporozoite prevalence (86.4%) and intensity (median 25,000) (Fig. 5d). Another Q_o_-site mutant, V284L, also showed a substantial decrease in oocyst prevalence (80.1% reduction) and intensity (90.9% reduction) relative to controls (Extended Data Table 2 and Extended Data Fig. 5), and we did not test for sporozoites in the salivary glands. The M133I atovaquone-selected CytB Q_o_-site mutant also produced fewer parasites (13.8% and 45.2% reduction in oocyst and sporozoite prevalence, respectively) (Extended Data Table 2). Therefore, even if CytB mutants emerged under double Q_o_–Q_i_-site selection, they would not be easily transmissible because of severe fitness costs that the parasites face during the mosquito stage of development. We did not test transmissibility of the Q_i_-site mutants in this study owing to either low resistance levels relative to the wild type (H12Q) or because the mutation is in a non-transmissible Dd2 genetic background (I22L).

## Discussion

Mosquito-targeted interventions aimed at killing *Plasmodium* parasites in *Anopheles* female mosquitoes represent an attractive and untapped area for malaria control. In asexual blood-stages, high-throughput in vitro screens have enabled the testing of millions of compounds for antimalarial activity^38–40^. Combined with genome-wide genetic studies^41,42^, these screens have identified thousands of genes that are essential to the parasite, many of which could be potential drug targets. However, similar studies of *Anopheles* female mosquitoes are hindered by the lack of a robust in vitro culture system^43^ despite recent advances^12^, and by the paucity of genetic tools that are suitable for those development stages. Because of these limitations, our screen was performed entirely in vivo after a careful selection of targets that we reasoned may be essential for parasite development in *Anopheles*. Our library included some compounds that are in the pipeline for possible development as human therapeutics, as we wanted to test as many targets as possible. Indeed, at some stage during their testing, these compounds may prove unsuitable for human use for reasons such as unfavourable pharmacokinetics, inadequate safety profiles or high likelihood of resistance, and therefore could be repurposed for use in mosquito-targeted interventions. This strategy could be particularly useful for clinically developed antimalarials that ultimately cannot be used as treatments in humans.

Resistance is always a concern when deploying new chemotherapeutics. Here we used an inhibitor, ELQ-453, that targets the same site as atovaquone and could therefore potentially induce mutations that lead to resistance to this prophylactic antimalarial. However, some considerations provide a reason for moderate optimism that parasite resistance would not rapidly emerge when using ELQ combinations. Targeting *Plasmodium* during a bottleneck in its life cycle—wherein there are often only a few tens of ookinetes and single-digit numbers of oocysts compared with the billions of asexual parasites present in an infected individual^11^—would substantially reduce the likelihood of de novo emergence of resistance-conferring mutations^44^. A combination therapy targeting two different sites in CytB would further mitigate the chances of emergence and spread of resistant mutants. Consistent with previous work^33,45^, we did not find cross-resistance between Q_o_-targeting or Q_i_-targeting compounds, which suggests that these inhibitors could be used effectively for combination therapy in *Anopheles*. Moreover, if resistance were to arise, mutants would most probably exhibit severe impairments in development at the mosquito stage. Indeed, several investigators (as well as this study) have reported that Q_o_-site mutants, including the clinically relevant Y268S mutation, have little to no transmission by *Anopheles* female mosquitoes^35–37^. This effect is probably due to compromised mitochondrial activity in an environment where parasites have a greater reliance on canonical mitochondrial oxidative phosphorylation and ATP generation than in the asexual blood stages. Notably, despite repeated attempts, we were unable to select for resistance to our most active Q_i_-site inhibitor, consistent with previous reports showing that in vitro resistance propensity of the Q_i_-site inhibitor ELQ-300 is low^32,33^. When combined, these results indicate that a dual-site combination of ELQs would reduce the risk of resistance, which would be further mitigated by careful monitoring of the emergence of resistance. The use of different antiparasitic compound combinations in humans and mosquitoes would also decrease the chances of the spread of resistant mutants to current and future therapeutics. As such, this approach could mitigate one of the major concerns in efforts to curb malaria. In future studies, it would be ideal to prioritize compounds that are active against mosquito stages but do not share a target with clinically used antimalarials. This strategy would both protect antimalarial treatment regimens from possible resistance selection in the mosquito and ensure that selection of resistant genotypes during treatment of patients does not jeopardize the efficacy of mosquito-targeted antiplasmodials. Such compounds would be well suited to reduce malaria transmission and extend the efficacy of existing bed nets and prevent the transmission of resistance against classical curative antimalarials.

In addition to essential, druggable targets, the success of mosquito-targeted transmission-blocking strategies will depend heavily on product stability and longevity, manufacturing scalability, affordability and community buy-in. Our polyethylene prototypes demonstrate that the top candidates identified in this screen preserve their activity in bed net-like materials and show promising longevity, activity in insecticide-resistant mosquitoes and long duration of effects. The additional impact on oocyst growth, which significantly delays sporozoite invasion of salivary glands and thereby extend the parasite EIP, would further reduce the mosquito vectorial capacity given the short lifespan of mosquitoes (2–3 weeks in field settings)^27–29^.

Notably, we performed a simplified, scalable three-step ELQ synthesis with relatively inexpensive starting materials. This straightforward synthesis is a promising indication of cost-effectiveness, and we anticipate that with additional process chemistry optimization, manufacturing-scale synthesis and bulk net production and procurement, these compounds will be an affordable and effective addition to bed nets even in the current highly competitive market. Advances in bed net designs in recent years have demonstrated that active ingredients can be used in a parsimonious way while preserving activity, such as by incorporating them in roof panels^46,47^ where mosquitoes are known to interact with nets the most^48^. ELQs could similarly be strategically used to minimize additional costs while maintaining parasite killing efficacy. Significant work remains, but at a time when insecticide resistance is recognized as a major threat to control efforts^1^, realizing the full potential of mosquito-targeted antiparasitic interventions may provide a new tool to reduce malaria transmission and overall disease burden.

## Methods

### Mosquito rearing

*A.* *gambiae* G3 mosquitoes were housed in cages in an insectary maintained at 27 °C with 70–80% humidity and a 12-h light–dark cycle. Colony cages were provided with water, 10% w/v glucose solution ad libitum and donated human blood (Research Blood Components) once a week for colony maintenance. For the experiments described in this article, G3 pupae were collected and placed into cages for eclosion, after which adult mosquitoes were provided with water and a 10% w/v glucose solution until the time of experimentation. All experiments were performed with age-matched female mosquitoes ranging from 4 to 7 days old.

### *P.falciparum* culture and gametocyte induction

Wild-type NF54 *P.* *falciparum* (used in accordance with a material-transfer agreement from the laboratory of C. Barillas-Mury) was used for all experiments in this article, with the exception of the following experiments: (1) the Dd2 CytB I22L and TM90-C2B CytB Y268S mutant lines (both courtesy of the laboratory of M. Riscoe) used for cross-resistance analysis; and (2) the CytB V259L, V284L and M133I resistant NF54 lines generated through ELQ selection. Wild-type and mutant NF54 cell lines were authenticated by whole-genome sequencing. Dd2 I22L was authenticated by Sanger sequencing^33^, and TM90-C2B is a clinical isolate^34^. All cell lines were tested for mycoplasma contamination, and TM90-C2B was mycoplasma-positive (all others were negative). Asexual blood-stage parasites were maintained between 0.2 and 2% parasitaemia in RPMI 1640 with l-glutamine and 25 mM HEPES medium (Corning) supplemented with 10 mg l^–1^ hypoxanthine, 0.2% sodium bicarbonate and 10% heat-inactivated human serum (Interstate Blood Bank) and 5% haematocrit in human red blood cells (Research Blood Components) in incubators kept at 37 °C and a gas composition of 5% O_2_, 5% CO_2_ and balanced N_2_ for up to 2 months per established protocols^49^. Gametocytes were induced by increasing the parasitaemia to >2% and incubating for 14–20 days with daily medium changes to accumulate stage V gametocytes per established protocols^50^.

### *A.gambiae* infection with *P.falciparum*

*A.* *gambiae* G3 mosquitoes (aged 4–7 days) were fed on 14–21-day *P.* *falciparum* NF54 induction cultures containing stage V gametocytes kept at 37 °C in custom glass, water-jacketed membrane feeders. Mosquitoes were allowed to feed for up to 60 min and then were placed into a custom-made infection glovebox for biosafety containment (Inert Technology). Any mosquitoes that did not fully engorge were aspirated into 80% ethanol and removed from subsequent experimentation. Mosquitoes were provided with water and a 10% w/v sugar solution to feed ad libitum for the duration of the experiment, and the room was kept at 27 °C with 70–80% humidity on a 12-h light–dark cycle.

### Topical applications

DMSO stocks (100 mM) were prepared for each compound in the topical-application screening library and stored in individual aliquots at −20 °C. On the day of topical application, DMSO stocks were thawed and diluted 1:50 in acetone to make the final 2 mM and 2% v/v DMSO working solution. A 2% DMSO control solution was also prepared in this manner. We chose this screening concentration to maximize the amount of compound applied to mosquitoes while minimizing the DMSO concentration. For compounds that were insoluble at 100 mM DMSO, 2 mM working solutions were instead prepared directly from powder stock with 2% v/v DMSO and acetone. In rare cases (noted in Supplementary Table 1), for compounds that were insoluble at 100 mM DMSO, working solutions were instead prepared from a saturated DMSO stock to maximize the final screening concentration (at a maximum solubility limit of less than 2 mM). Female *A.* *gambiae* G3 mosquitoes (aged 4–7 days) were anaesthetized on ice and 0.5 µl working solution or DMSO control was pipetted directly onto the dorsal thorax. Mosquitoes were removed from the ice and placed into a cage to recover for 4–6 h before being provided an infectious blood meal. All compounds were screened once, and compounds that showed a preliminary reduction in parasite prevalence were assayed at least once more in a second biological replicate to confirm activity.

### Tarsal-contact assays

Tarsal-contact assays were performed as previously described^3^, but with some modifications. Tarsal-contact plates were prepared by calculating the amount of compound necessary to coat a 6-cm-diameter glass Petri dish (0.02827 m^2^ total surface area) at 1 mmol m^–2^. This concentration is roughly 10× the previously described atovaquone tarsal-contact EC_99_ value^9^, and we reasoned that this high concentration would enable detection of active compounds. Powder compounds were weighed out and solubilized in acetone and diluted as necessary to reach the calculated surface-area concentration. Next, 100 mg m^–2^ of the adjuvant Mero (also known as rapeseed methyl esters and emulsifier ethoxy (7) tridecanol (RME), a gift from H. Ranson) was also added to each solution, which has previously been shown to act as an uptake excipient in mosquito tarsal-contact assays^51^. For ELQ-453–ELQ-613 combination exposure, solutions were prepared so that the total compound concentration was 1 mmol m^–2^ made up of 0.5 mmol m^–2^ ELQ-453 and 0.5 mmol m^–2^ ELQ-613. Stock solutions were serially diluted to prepare lower concentration plates for tarsal contact dose–response curves. The final 1 ml acetone solution containing 100 mg m^–2^ RME and the compound at the desired concentration was pipetted onto a 6 cm glass Petri dish. A 100 mg m^–2^ RME control plate was also prepared. The plates were left at room temperature overnight on an orbital shaker to fully evaporate the acetone. The next day, plates were fitted with a transparent plastic lid to contain mosquitoes during exposure. Female *A.* *gambiae* G3 mosquitoes (4–7 days old) were mouth aspirated into the exposure apparatus for 6 min. A maximum of 25 mosquitoes were exposed at a time to minimize crowding and to allow all mosquitoes to come into contact with the drug-coated or vehicle-coated surface. In each experiment, mosquitoes were visually monitored to confirm tarsal contact with the coated surface. We did not observe obvious excessive flight or other apparent indications of avoidance behaviour, although this was not empirically recorded. After 6 min, mosquitoes were released into a cage and provided with an infectious blood meal 1 h later. For post-infection tarsal contact, exposures were performed as previously described^4^. At 3 days after an infectious blood meal, mosquitoes were transferred into the exposure apparatus as described above, and after 6 min of tarsal-contact exposure, they were transferred to a new cage by mouth aspiration. For thin-film and dipped-net tarsal contact, exposures were performed as described above with 6-cm-diameter plastic lids fitted to the polyethylene thin films or the dipped net to contain mosquitoes.

### Net-dipping protocol

White polyester netting (12.5 × 12.5 cm) was used for tarsal-contact assays on nets. The netting was initially soaked in acetone and then allowed to air dry for 30 min. After this time, netting was soaked in equivalent amounts of ELQ-453 and ELQ-613 in acetone or in acetone alone (control) to achieve the desired ELQ mg m^–2^ concentration and rocked until the entire solution was absorbed. Netting was allowed to air dry overnight on a flat surface, and tarsal-contact assays were performed the next day.

### Oocyst dissection and quantification

For all oocyst data, at 7 d.p.i., mosquitoes were aspirated into 80% ethanol, incubated at –20 °C for 10 min, then transferred to 1× PBS at room temperature for dissection. Midguts were dissected into 1× PBS, stained by transferring into 0.2% w/v mercury dibromofluorescein disodium salt solution (mercurochrome, Sigma-Aldrich) in ddH_2_O for 14 min and mounted in 0.02% w/v mercurochrome for microscopy. Midguts were imaged and oocysts counted at ×100 magnification on an Olympus Inverted CKX41 microscope. For post-infection experiments, oocyst size (cross-sectional area) was measured using the ImageJ software fork Fiji^52^.

### Sporozoite dissection and quantification

At 11 or 14 d.p.i., mosquitoes were aspirated from their cages in the infection glovebox into 80% ethanol, incubated at −20 °C for 10 min, then transferred to 1× PBS at room temperature for dissection. Mosquito heads were removed and salivary glands from individual mosquitoes were dissected and collected in 50 µl 1× PBS. Salivary gland tissue was mechanically disrupted with a small handheld pestle and 10 µl was loaded onto a haemocytometer. Sporozoites were counted using an Olympus Inverted CKX41 microscope at ×100 magnification with phase contrast, and the total number of sporozoites per mosquito was calculated.

### Live ookinete IFAs

Cipargamin, M5717, ELQ-613 or 2% DMSO as control was topically applied to mosquitoes before an infectious blood meal as described above. At 22 h.p.i., 10 mosquitoes per group were aspirated into 80% ethanol, incubated at −20 °C for 10 min, then transferred to 1× PBS at room temperature for dissection. Midguts were dissected with their blood bolus intact into 1.5 ml low-bind Eppendorf tubes containing 100 µl 1× PBS. The midgut tissue was disrupted by gently pipetting midguts up and down approximately 20 times until the blood bolus was visibly released and midguts were no longer intact. This homogenate was centrifuged at 300*g* for 5 min and the supernatant was discarded. The pellet containing homogenized blood and midgut tissue was resuspended in 100 µl of 1× PBS with 1:300 mouse anti-PfS25 (BEI Resources) primary antibody conjugated to CF-488 dye (Sigma-Aldrich) and 1:500 Hoechst (AAT Bioquest) and incubated in the dark at −4 °C for 30 min. After incubation, samples were again spun down at 300*g* for 5 min and resuspended in 100 µl 1× PBS wash. Samples were spun down again and resuspended in a final volume of 10 µl 1× PBS, which was mounted onto a microscope slide (VWR) and imaged on an inverted Zeiss Axio Observer Z1 at ×200 magnification. Approximately 200 parasites per group were live counted and classified as a zygote, an intermediate retort form or an ookinete on the basis of parasite morphology.

### Sample preparation for MS analysis

Female mosquitoes were exposed to test compounds (100 µmol m^–2^ ELQ-453, ELQ-456, ELQ-458, ELQ-613, ELQ-614, ELQ-618 and ELQ-465) through 6 min of tarsal contact as described above, transferred to new cages and provided with a continuous supply of water and 10% w/v glucose solution until the end of the experiment. Midguts from treated mosquitoes were then collected at the following time points after tarsal-contact exposure: 1, 3, 6, 24 and 48 h. Ten mosquitoes per time point were aspirated into 80% ethanol, transferred into PBS on ice and dissected. For each time point, 5 whole midguts were collected and pooled into 1.5 ml Eppendorf tubes containing 400 μl methanol and a 2 mm glass bead, resulting in 2 technical replicates per time point. Two biological replicates (with two technical replicates each) were performed in total. Samples were stored at −80 °C until homogenization.

ELQs with similar structures to the test compounds were used as internal standard (IS) samples for detection and quantification: prodrug ELQ-615 and its active form ELQ-183, and prodrug ELQ-331 and its active form ELQ-300. An IS master mix was prepared with 2 nM of each standard in methanol. Next, 100 μl of this IS master mix was added to each sample to obtain a total final volume of 500 μl. Samples were homogenized through bead beating in a cold block at 2,400 r.p.m. for 1.5 min, 3 times. After homogenization, the samples were centrifuged for 10 min at 15,060 r.p.m. at 4 °C. The supernatant, containing the extracted material, was carefully transferred to new 1.5 ml Eppendorf tubes. Samples were kept on ice throughout the extraction process. The prepared supernatants were stored at −80 °C until submission to the Harvard Center for MS analyses.

### MS analysis

All solvents were LC–MS grade (Millipore Sigma). Samples were evaporated to dryness under nitrogen flow and resuspended in 20 µl methanol 80% in water. Samples were quantified using an ABSciex 7500 triple quadrupole mass spectrometer (AB Sciex) coupled to an 1290 Agilent LC (Agilent). Five microlitres of the sample was injected into a Kinetex C18 column (2.1 mm × 150 mm, Phenomenex) maintained at 55 °C. The mobile phases were A (water and 0.1% formic acid) and B (acetonitrile and 0.1% formic acid). The following gradient was used: 10% B for 5 min, then to 100% B in 13 min, maintained at 100% B for 4 min, followed by 2 min re-equilibration at 10% B. The flow rate was 0.3 ml min^−1^. The instrument was operated in positive mode with the following source conditions: gas 1 at 40 psi, gas 2 at 65 psi, curtain gas at 44, collision (CAD) gas at 8, temperature at 525 °C and spray voltage at 1,800 V. See Supplementary Table 3 for the multiple reaction monitoring transitions used. A shorter cleaning method was used to inject a solvent blank between each sample. For the standard curve, a series of solutions in methanol were prepared, with the highest at 500 nM for all targets and 12 successive one-third dilutions. One hundred microlitres of each solution was added to unexposed midgut homogenates. The standard curve samples were then processed as for the samples. Targets were quantified using the area under the curve for the quantifier transitions divided by the area of the quantifier of the corresponding internal standards using Sciex OS Analytics (AB Sciex).

### Asexual blood-stage parasite culture for resistance selection

NF54 parasite lines (obtained through a material-transfer agreement from the laboratory of C. Barillas-Mury) were grown at 4–5% haematocrit in fresh human erythrocytes (O^+^) in RPMI 1640 medium supplemented with 10% O^+^ human serum (heat-inactivated and pooled), 26.6 mM NaHCO_3_, 27.7 mM HEPES, 0.41 mM hypoxanthine and 25 µg ml^–1^ gentamicin. Human serum and erythrocytes were supplied by Interstate Blood Bank. Cultures were incubated at 37 °C under 1% O_2_, 5% CO_2_ and balance N_2_ gas.

### Selection of CytB mutants

To generate CytB mutants, NF54 parasites were exposed to various CytB inhibitors as follows. Parasites were exposed to 2 nM atovaquone (M133I), 5 nM or 10 nM ELQ-121 (V259L), 5 nM or 10 nM ELQ-400 (V259L and V284L, respectively), or 2 nM or 10 nM for ELQ-437 (V259L and Y126C mixed) and kept under constant drug pressure until parasites recurred. For ELQ-300, parasites were incubated with 50 nM ELQ-300 (H12Q) for 5 weeks, after which drug pressure was removed to let parasites recover. Mutations were identified by amplification of the CytB-encoding gene (*MT-CYB*) by PCR (primers: *MT-CYB* 5′ UTR: GGATGGAATATGATTTGTTCTATTGGG and mitoR: TTATATGTTTGCTTGGGAGC) and subsequent Sanger sequencing, except for the H12Q *MT-CYB* Q_i_-site mutation, which was identified by whole-genome sequencing as described below.

### Whole-genome sequencing of CytB mutants

Parasite samples from parental-susceptible NF54 and selected mutants were collected from asexual blood-stage cultures and from gametocyte cultures directly before infectious blood meals. Parasite DNA was extracted using DNeasy Blood & Tissue kits (Qiagen) for whole-genome sequencing. Sequencing libraries were prepared by the University of California San Diego Institute for Genomic Medicine Genomics Center using a Nextera XT kit (FC-131-1024, Illumina) with 2 ng input gDNA and standard dual indexing.

Raw sequencing reads were aligned to the *P.* *falciparum* 3D7 reference genome^53^ (PlasmoDB v.13.0) and preprocessed following standard GATK (v.3.5) protocols. GATK HaplotypeCaller was used to call single-nucleotide variants and insertions and deletions, which were hard filtered based on the basis of the following exclusion criteria: ReadPosRankSum >10.0 or <–10.0; quality by depth < 1.5; or mapping quality rank sum (MQRankSum) <–14. Variants were annotated using SnpEff with a custom database built from the 3D7 reference GFF from PlasmoDB (v.13.0). Fisher’s exact test comparing reference versus alternate allele counts in each sample to its parent was used to compute a *P* value for each variant call to assist in determining whether it significantly differed from the drug-sensitive parent at the same genomic locus.

Copy number variations (CNVs) were detected from whole-genome sequencing data by calculating denoised log_2_ copy ratios across gene intervals using the GATK 4.0 CNV pipeline. Read counts for each sample were computed across a predefined gene interval list with intergenic regions and the highly variable antigenic *var*, *rifin*, *stevor* and *surfin* genes were removed. Denoising was performed against a panel of normals consisting of 30 independently sequenced 3D7 parent clones (excluding parental lines in this study). CNVs were retained if at least 4 sequential genes showed a denoised log_2_ copy ratio of at least 0.6, which indicated gene amplification, or at most −0.6, which indicated a deletion. ELQ121-NF54-R-ABS was excluded from CNV analysis owing to extreme variability in copy ratios across the genome. No CNVs in core regions were found using this method.

### In vitro drug susceptibility assays by SYBR Green I staining

Drug susceptibility assays were performed as previously described using the SYBR Green I method^54^. In brief, synchronized rings at 1% parasitaemia and 1% haematocrit in 40 µl of 0.5% serum-complimented medium were grown for 72 h in 384-well clear bottom plates in the presence of different drug concentrations. All drug conditions were performed in three technical replicates, with at least three biological replicates. Drugs were dispensed in 12-point dilution series into 384-well plates by aa HP D300e Digital Dispenser (Hewlett Packard). Growth at 72 h was quantified by staining parasite DNA with SYBR Green I (Lonza) for 24 h and measuring relative fluorescence units at an excitation of 494 nm and an emission of 530 nm using a SpectraMax M5 (Molecular Devices). EC_50_ values were calculated using a nonlinear regression with the log(inhibitor) versus response–variable slope curve-fitting algorithm in GraphPad Prism (v.10; GraphPad Software).

### Statistics

All statistical analyses were performed in GraphPad Prism (v.10.0; GraphPad Software) and JMP Pro 17 (SAS Institute). Experimental sample sizes, randomization and blinding were based on previously published protocols^3,4^ and are described in detail in the Reporting Summary. For topical screening and secondary tarsal screening, the control versus compound-treated infection prevalence two-sided OR and 95% CI values were calculated using the Baptista–Pike method. The OR and 95% CI were plotted, and any treatment group with an upper confidence interval of <1 was considered a significant reduction in infection prevalence. No compounds were found to significantly increase infection prevalence. In the case of two compounds (DSM161 and DSM703), both the control and compound-treated group had 100% prevalence. In this case, two out of the four cells in the contingency table were 0 and the OR could not be calculated. We therefore arbitrarily added 1 to the uninfected cells for each group to calculate and plot an estimated OR. We similarly added 1 to the uninfected MMV019881 contingency table cell, as otherwise the calculated OR upper CI was infinity. For infection experiments, prevalence was analysed using two-tailed Fisher’s exact tests, and intensity was analysed using two-tailed Mann–Whitney tests. In experiments for which more than two groups were compared, prevalence was analysed using Fisher’s exact tests followed by Bonferroni correction, and intensity was analysed using Kruskal–Wallis tests with Dunn’s post hoc multiple-comparisons correction. For 22 h.p.i. IFAs, the numbers of each stage of parasite development (1, zygote; 2, retort; 3, ookinete) detected in midguts (1 pool of 10 midguts; 5 biological replicates) of treated mosquitoes at 20–22 h.p.i. were analysed using an ordinal logistic regression model in JMP Pro 17 (SAS Institute), incorporating ‘treatment’ (control (DMSO), cipargamin, M5717 or ELQ-613) and ‘mosquito sample’ (nested within treatment).

### QSAR analysis of topical activity

An initial set of 38 compounds was used for QSAR modelling (Supplementary Table 2). This set included compounds that shared the mechanism of action with the selected hits in the topical screen and atovaquone, which showed full activity (topical OR = 0). The OR topical mean value (dependent variable) was transformed according to the following formula: 1 – [log(OR topical) + 1)]. This formula allows activity values to fall between 0 (lowest activity) and 1 (highest activity). The molecular operating environment (MOE) software (v.2022.02; Chemical Computing Group) was used to prepare the library, calculate descriptors and run the QSAR analysis. In MOE, molecules were washed to their dominant ionization form at pH 7 and energy minimization was applied to generate the three-dimensional structure from the two-dimensional canonical SMILES. The compound collection was subdivided into 27 chemical clusters (85% similarity) using MACCS fingerprint. Based on their activity values and chemical clusters, molecules were evenly distributed into training set (85%, 32 compounds) and test set (15%, 6 compounds) (Supplementary Table 2). A total of 347 molecular descriptors (209 i2D descriptors and 138 i3D descriptors) were computed. Descriptors that correlated better with the dependent variable were selected using the contingency module (196 descriptors). Three model types were built using the AutoQSAR and QSAR-Evolution codes of MOE: partial least dquare (PLS), principal component regression (PCR) and genetic algorithm–multiple linear regression (GA-MLR). To prevent model overfitting during model generation, low variance (threshold 0.9) descriptors were excluded, and the number of descriptors in the model was limited to six. Outliers (*z*′ score limit of 2.5) were excluded during model building. Predictive precision and goodness-of-fit of each model was assessed using the coefficient of determination (*R*^2^) and root mean square error (RMSE). To evaluate overfitting, cross-validation was run using the leave-one-out cross-validation (LOO-CV) method, giving ${R}_{{\rm{CV}}}^{2}$ and RMSE_CV_. External validation on the test set was assessed with correlation coefficient ${R}_{{\rm{EXT}}}^{2}$. The best model obtained for topical activity was model GA-MLR, whereas PLS and PCR had poor fitting and did not satisfy the threshold external validation parameters. Correlation plot graphs comparing observed and predicted values were plotted using GraphPad Prism (v.10.0; GraphPad Software).

### Compound library evaluation through CACTI

SMILES for each compound tested in this study were saved as a tabular file and queried using CACTI^55^ command line version and Python 3.5 (Supplementary Table 4). The search was performed to obtain analogues (flag -simnet -sdb) with 90% similarity threshold, as well as literature references (flag -lit -slit) for both the compound library and analogues. Chemical names and SciFinder CAS identifiers (numerical series) found by CACTI were extracted from the results of synonyms and inputted as an additional column for easier access. Compounds with missing entries were obtained using ChemDraw 22.2 and the SciFinder CAS-n web tool when available. Public references obtained with CACTI were manually confirmed, and cases in which CACTI found evidence of a target different from our study are indicated. Analogues with a validated target were used for validation and the rest filtered to reduce the complexity of the CACTI analysis.

### ELQ synthesis

Synthetic methods and small-molecule characterization are provided in the Supplementary Information and Supplementary Table 5.

### Preparation of ELQ LDPE films

LDPE polymer (0.5 g) and 25 ml xylenes were combined in the lid of a 6 cm glass Petri dish and heated at 150 °C until the polymer dissolved. It was necessary to occasionally replenish evaporated xylenes. ELQ in xylenes was then added to this solution. Excess xylenes were evaporated slowly and the resulting crude polymer films were allowed to dry overnight. The bottom of the 6 cm glass Petri dish was then placed against the crude polymer thin film. The assembled apparatus was then heated on a hotplate with a weight placed against the bottom of the Petri dish. The resulting ELQ-containing extruded polymer thin-film was allowed to cool and peeled off the Petri dish lid using a laboratory spatula.

### Preparation of extruded ELQ HDPE films

HDPE pellets (McMaster-Carr, 7202N11) were cryogenically milled into a flowable powder by freezing the pellets with liquid nitrogen and then milling the cold HDPE through a Retsch Mill with a 1.0 mm sieve to maximize blending efficiency and uniformity and to increase the rate of melting of the HDPE. The dried HDPE powder and ELQ powders were weighed, combined and physically mixed until uniform. HDPE powder and ELQ mixtures were placed into a feeder and fed into a Haake MiniLab (Thermo Electron) twin screw extruder with a barrel temperature of 150 °C. The blend was extruded through a 3-mm-diameter nozzle, collected and allowed to cool in ambient conditions. The collected filaments for each formulation were stored in a sealed bag until use. Each of the collected formulation filaments was cut to sizes of roughly 5–7.5 cm in length. The platens on a Model C, Carver press with two stainless steel sheets covered in aluminium foil and a stainless steel spacer (with a square cavity of 10.16 cm × 10.16 cm × 0.08 cm thick) were preheated to 150 °C. The plates were then removed from the press and 7.5 g of the 5–7.5 cm cut filament were placed in the middle of the plates (with one plate on the bottom and insert around the filaments and the second plate on top of the filaments). The mould was placed back into the Carver press and gently compressed for 1.5 min. The sample was then pressed to 2267.962 kg (5,000 lbs) for 1 min and 4535.924 kg (10,000 lbs) for 30 s. The plates were removed from the Carver press and allowed to cool in ambient conditions for 15 min with a weight on top of the mould. The films were deburred and stored in Ziploc bags until tarsal-contact assays were performed.

### Ethics statement

Human whole blood was ethically sourced from healthy volunteers, and their research use was in accord with the terms of the informed consent under a protocol approved by the Institutional Review Board (IRB 120160613, Research Blood Components).

### Reporting summary

Further information on research design is available in the Nature Portfolio Reporting Summary linked to this article.

## Online content

Any methods, additional references, Nature Portfolio reporting summaries, source data, extended data, supplementary information, acknowledgements, peer review information; details of author contributions and competing interests; and statements of data and code availability are available at 10.1038/s41586-025-09039-2.

## Supplementary information

Supplementary Information This file contains a description of the ELQ synthesis methods and chemical characterization, legends for supplementary tables and supplementary references.

Reporting Summary

Supplementary Tables Supplementary Tables 1–5.

## Source data

Source Data Fig. 1

Source Data Fig. 2

Source Data Fig. 3

Source Data Fig. 4

Source Data Fig. 5

Source Data Extended Data Fig. 1

Source Data Extended Data Fig. 2

Source Data Extended Data Fig. 3

Source Data Extended Data Fig. 4

Source Data Extended Data Fig. 5

## Figures and Tables

**Extended Data Table 1:**
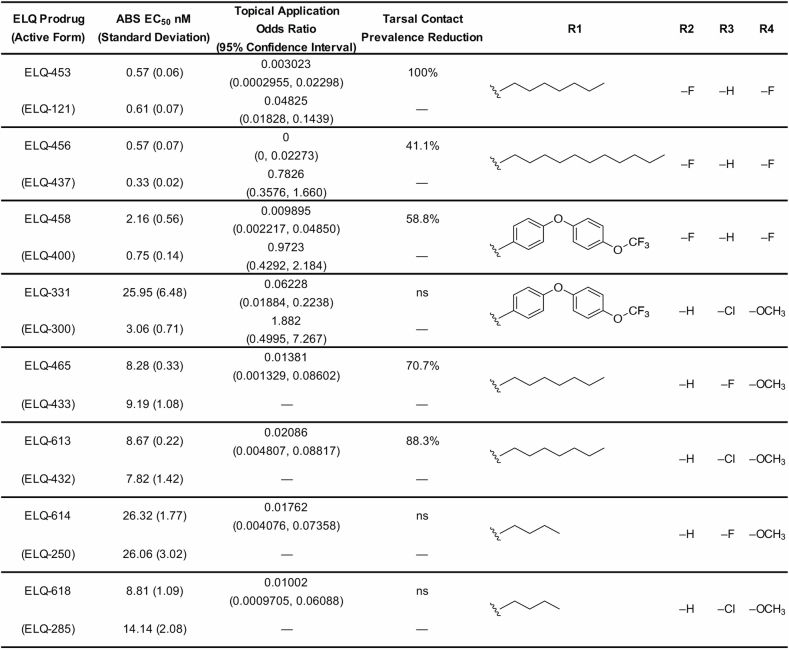
Endochin-like quinolone activity and structure

ELQ compound activity against WT *P. falciparum* NF54 in asexual blood stages (ABS) and mosquito stages. ABS half maximal effective concentrations (EC_50_) were calculated from 72 h drug susceptibility assays via nonlinear regression. Average EC_50_s from three biological replicates are shown with standard deviations reported in parentheses. Topical application odds ratios of compound treated versus DMSO control infection prevalence are given, with 95% confidence intervals noted in parentheses. Odds ratios and 95% confidence intervals were calculated by the Baptista-Pike method and compounds with an upper bound confidence interval below 1 significantly reduced infection prevalence relative to control. Tarsal contact percent reduction in prevalence relative to control (based on prevalence data in Fig. 3b-c, two biological replicates). Modifications to the core endochin-like quinolone structure for each prodrug and active form. Core ELQ structure and positions of each R group shown in Fig. 3a.

**Extended Data Table 2:**
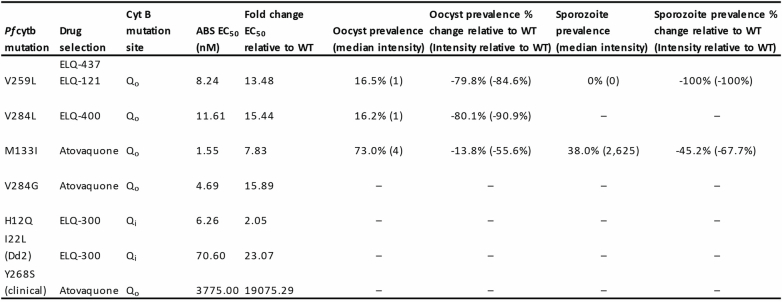
CytB mutant resistance and transmissibility

*P. falciparum* CytB mutants were generated in NF54 asexual blood stages (ABS) via selection with Q_o_-site inhibitors ELQ-121, ELQ-400, ELQ-437, and atovaquone, and Q_i_-site inhibitors ELQ-300. Previously established CytB mutant lines were also used: the I22L Q_i_-site mutant in a non-transmissible *P. falciparum* Dd2 background and the Y268S Q_o_-site mutant clinical *P. falciparum* isolate. V259L was independently selected with both ELQ-437 and ELQ-121, but follow up experiments were performed with the ELQ-121 selected line. ABS half maximal effective concentrations (EC_50_) were calculated from 72 h drug susceptibility assays and the fold-change in EC_50_ of each mutant line relative to WT *P. falciparum* NF54 is reported. Mosquito transmission experiments were performed with the V259L, V284L, and M133I lines. Oocyst prevalence at 7 days post-infection (dpi) and sporozoite prevalence 14 dpi are reported with median parasite intensities in parentheses. Percent change of mutant oocyst and sporozoite prevalence and intensity relative to WT is reported.

## Data Availability

Whole-genome sequencing data have been deposited in the NCBI Sequence Read Archive (BioProject accession: PRJNA1121409). The *P.* *falciparum* 3D7 reference genome^53^ (PlasmoDB v.13.0) is available at BioProject accession PRJNA13173 (v.13 FASTA: https://plasmodb.org/common/downloads/release-13.0/Pfalciparum3D7/fasta/data/PlasmoDB-13.0_Pfalciparum3D7_Genome.fasta). All other data supporting the findings of this study are available in the article, its Supplementary Information and source data available from the Harvard Dataverse repository (10.7910/DVN/UFRFAG). Source data are provided with this paper.
