# Supplementary material for: In vivo screen of Plasmodium targets for mosquito-based malaria control
Source: Nature. 2025 May 21;643(8072):785–93. doi: 10.1038/s41586-025-09039-2 (PMC12267055; doi:10.1038/s41586-025-09039-2)
Supplement: Supplementary file 1 — This file contains a description of the ELQ synthesis methods and chemical characterization, legends for supplementary tables and supplementary references. [file 41586_2025_9039_MOESM1_ESM.docx]

**Supplementary Information**

**Supplementary Methods**

*General Chemistry.* All chemicals were purchased from Combi-Blocks, San Diego, CA (USA), Fisher Scientific, Hampton, NH (USA), Oakwood Chemical, Estill, SC (USA), Sigma-Aldrich Chemical Company in St. Louis, MO (USA), or TCI America, Portland, OR (USA) and were used as received. An Optimelt Automated Melting point system from Stanford Research Systems, Sunnyvale, CA (USA) was used to obtain melting points. Analytical TLC was performed using DC Kieselgel 60F_254_ precoated silica gel plates and visualized under 254 nm UV light. An Agilent Technologies (Santa Clara, CA, USA) 7890B gas chromatograph (30 m, DBS column set at either 100 °C or 200 °C for 2 min, then at 30 °C/min to 300 °C with inlet temperature set at 250 °C) with an Agilent Technologies 5977A mass-selective detector operating at 70 eV was used to obtain GC-MS data. An Isolera One flash chromatography system from Biotage (Uppsala, Sweden) was used to perform silica gel flash chromatography with methods derived from TLC results. A Bruker (Billerica, MA) 400 MHz Avance NEO NanoBay NMR spectrometer operating at 400.14 MHz was used to obtain NMR spectra. NMR data were analyzed using iNMR (Nucleomatica, Molfetta, Italy) Spectrum Analyst software. NMR chemical shifts were reported in parts per million (ppm) relative to internal tetramethylsilane (TMS) standard or residual solvent peak, and coupling constant values (*J*) were reported in hertz (Hz). For fluorine containing compounds, coupled ^19^F spectra operating at 376 MHz were obtained. Also, for fluorine containing compounds, additional ^13^C peaks were observed due to splitting by fluorine; fluorine coupling patterns were not reported. High-resolution mass spectrometry (HRMS) was performed using a high resolution (30,000) Thermo LTQ-Orbitrap Discovery hybrid mass spectrometry instrument (San Jose, CA) equipped with an electrospray ionization source operating in the positive or negative ion mode. The Orbitrap was externally calibrated prior to data acquisition allowing accurate mass measurements for [M + H]^+^ ions to be obtained within 4 ppm. All final compounds were determined to be greater than 95% pure by UV HPLC. Data was collected on an Agilent 1260 Infinity II HPLC with detection at 230 nm equipped with a Phenomenex (Torrance, CA, USA) Luna 5 μm C8(2) 100 Å reverse phase 50 × 2 mm LC column (order # 00B-4249-B0) at 40 °C and eluted with a gradient of A/B at 75:25% to A/B at 10:90% (A: 0.05% formic acid in water, B: 0.05% formic acid in methanol, both HPLC grade).

*
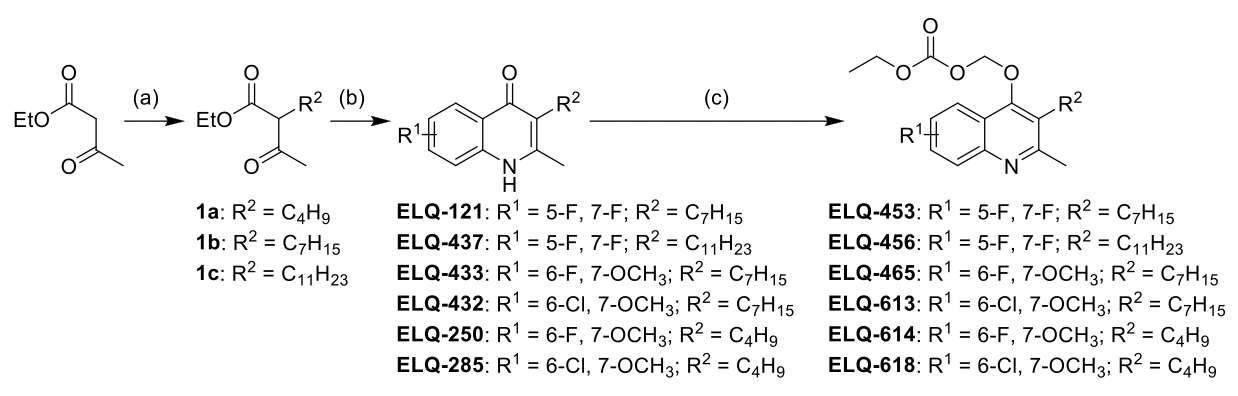
Supplemental Scheme 1: (a) ethylacetoacetate, sodium ethoxide, alkyl halide, ethanol, reflux, 24 hours; (b) 1. aniline, cat. HCl, benzene, reflux, 24-48 hours, 2. Dowtherm A, boiling, 10 minutes; (c) chloromethyl ethyl carbonate, tetrabutyl ammonium iodide, potassium carbonate and DMF, 60°C, 24 h.*

*General Procedure A for the synthesis of 4(1H)-quinolone ELQ:* A solution of ethyl acetoacetate (0.04 mol), sodium ethoxide (1 equivalent) and 1-bromoalkane (1 equivalent) in alcohol was refluxed for 24 hours (see Supplemental Scheme 1). The solvent was removed in vacuo, and the resulting residue was stirred with 150 ml of hexanes and filtered. The resulting filtrate was concentrated in vacuo to give crude **1a**, **1b** or **1c**, which were used without further purification. A mixture of crude **1a**, **1b** or **1c**, aniline (1 equivalent, calculated from ethyl acetoacetate used to prepare **1a**, **1b** or **1c**), benzene (0.45 molar) and catalytic concentrated *p*-toluenesulfonic acid was refluxed for 24-48 hours using a Dean-Stark trap to azeotropically remove the water formed during the condensation reaction. The reaction was complete when starting material was consumed (monitored by GC-MS). The reaction was concentrated in vacuo, and the resulting residue was added to 65 ml of boiling Dowtherm A (0.62 molar) over 4 minutes and boiled for another 10 minutes. After cooling, the product ELQ was isolated by filtration, washed with hexanes and recrystallized from aqueous alcohol.

*General Procedure B for the synthesis of the alkoxy carbonate prodrug:* A stirred mixture of 4-(1H)-quinolone **ELQ** (1 eq), tetrabutylammonium iodide (2 eq), chloromethyl ethyl carbonate (2 eq) and dry potassium carbonate (2 eq) in DMF was heated at 60°C until TLC showed no more starting material remained, usually over 24 hrs. The mixture was cooled to room temperature, filtered and the filtrate concentrated to dryness to give of an oil. The resulting residue was stirred with ethyl acetate for 30 minutes and the insoluble tetra-butyl ammonium iodide filtered and wash with ethyl acetate. The filtrate was concentrated to dryness and purified by flash chromatography using a gradient of ethyl acetate/hexane as eluent to give the desired prodrug. If the resulting prodrugs were not pure enough by TLC, GM-MS and NMR they can be obtained in pure form by recrystallization from hexane/ethyl acetate.

*5,7-Difluoro-3-n-heptyl-2-methylquinolin-4(1H)-one (ELQ-121).* Synthesized (see Supplemental Scheme 1) using a published procedure^1^.

*3-Butyl-6-fluoro-7-methoxy-2-methyl-4(1H)-quinolone (ELQ-250).* General Procedure A (see Supplemental Scheme 1). ^1^H-NMR (400 MHz; DMSO-d_6_): δ 11.33 (s, 1H), 7.65 (d, *J_F_* = 11.9 Hz, 1H), 7.02 (d, *J_F_* = 7.4 Hz, 1H), 3.92 (s, 3H), 2.46 (t, *J* = 7.4 Hz, 2H), 2.35 (s, 3H), 1.38-1.31 (m, 4H), 0.90 (t, *J* = 7.1 Hz, 3H). ^13^C-NMR (101 MHz; DMSO-d_6_): δ 174.97, 174.94, 150.95, 150.81, 150.3, 147.9, 145.7, 137.2, 118.8, 117.39, 117.34, 110.35, 110.16, 100.6, 56.5, 31.3, 24.9, 22.8, 17.9, 14.4. ^19^F-NMR (376 MHz; DMSO-d_6_): δ -140.1 (dd, J_H_ = 11.75, 7.53). HRMS (ESI) m/z for [C_15_H_18_FNO_2_]: Calculated [M+H]^+^: 264.1394, Found [M+H]^+^: 264.1390.

*3-Butyl-6-chloro-7-methoxy-2-methylquinolin-4(1H)-one (ELQ-285).* General Procedure A (see Supplemental Scheme 1). ^1^H-NMR (400 MHz; DMSO-d_6_): δ 11.35 (s, 1H), 7.96 (s, 1H), 6.99 (s, 1H), 3.93 (s, 3H), 2.45 (t, *J* = 7.4 Hz, 2H), 2.35 (s, 3H), 1.35-1.32 (m, 4H), 0.89 (t, *J* = 7.1 Hz, 3H). ^13^C-NMR (101 MHz; DMSO-d_6_): δ 174.6, 156.7, 146.0, 139.8, 126.4, 119.5, 118.3, 117.9, 99.7, 56.7, 31.3, 24.9, 22.8, 17.9, 14.4. HRMS (ESI) m/z for [C_15_H_18_ClNO_2_]: Calculated [M+H]^+^: 280.1099, Found [M+H]^+^: 280.1095.

*6-Chloro-3-n-heptyl-7-methoxy-2-methylquinolin-4(1H)-one (ELQ-432).* General Procedure A (see Supplemental Scheme 1). The crude product was boiled with methanol, filtered and subsequently re-crystallized from d.m.f. ^1^H-NMR (400 MHz; DMSO-d_6_): δ 11.35 (s, 1H), 7.96 (s, 1H), 6.99 (s, 1H), 3.93 (s, 3H), 2.44 (t, *J* = 7.5 Hz, 2H), 2.34 (s, 3H), 1.38-1.22 (m, 10H), 0.86 (t, *J* = 6.9 Hz, 3H). ^13^C-NMR (101 MHz; DMSO-d_6_): δ 174.6, 156.7, 146.0, 139.8, 126.4, 119.6, 118.3, 117.9, 99.7, 56.8, 31.8, 29.7, 29.16, 29.04, 25.2, 22.6, 17.9, 14.4. HRMS (ESI) m/z for [C_18_H_24_ClNO_2_]: Calculated [M+H]^+^: 322.1568, Found [M+H]^+^: 322.1565.

*6-Fluoro-3-n-heptyl-7-methoxy-2-methylquinolin-4(1H)-one (ELQ-433).* General Procedure A (see Supplemental Scheme 1). ^1^H-NMR (400 MHz; DMSO-d_6_): δ 11.34 (s, 1H), 7.65 (d, J = 11.9 Hz, 1H), 7.02 (d, J = 7.3 Hz, 1H), 3.92 (s, 3H), 2.44 (t, J = 7.2 Hz, 2H), 2.35 (s, 3H), 1.36−1.25 (m, 9H), 0.85 (t, J = 6.3 Hz, 3H). ^13^C-NMR (101 MHz; DMSO-d_6_): δ 175.0, 150.95, 150.81, 150.3, 147.9, 145.7, 137.2, 118.9, 117.39, 117.34, 110.34, 110.16, 100.6, 56.5, 31.8, 29.7, 29.16, 29.05, 25.2, 22.6, 17.9, 14.4. ^19^F-NMR (376 MHz; DMSO-d_6_): δ -140.1 (dd, J_H_ = 11.82, 7.44). HRMS (ESI) m/z for [C_18_H_24_FNO_2_]: Calculated [M+H]^+^: 306.1864, Found [M+H]^+^: 306.1860.

*5,7-Difluoro-2-methyl-3-undecylquinolin-4(1H)-one (ELQ-437).* General Procedure A (see Supplemental Scheme 1). ^1^H-NMR (400 MHz; DMSO-d_6_): δ 11.43 (s, 1H), 7.01-6.92 (m, 2H), 2.40 (t, J = 7.3 Hz, 2H), 2.33 (s, 3H), 1.37-1.24 (m, 18H), 0.85 (t, J = 6.9 Hz, 3H). ^19^F-NMR (376 MHz; DMSO-d_6_): δ -140.1 (dd, J_H_ = 12.11, 7.20). Because of insufficient solubility, we were not able to obtain ^13^C-NMR or HRMS data for **ELQ-437**. However, the identity of **ELQ-437** was verified by ^13^C-NMR or HRMS, because we were able to obtain ^13^C-NMR or HRMS data for a derivative, prodrug **ELQ-456**.

*((5,7-Difluoro-3-heptyl-2-methylquinolin-4-yl)oxy)methyl ethyl carbonate (ELQ-453).* Following the general Procedure B using **ELQ-121** (2.93 g, 10 mmol, 1eq), tetrabutylammonium iodide (7.38 g, 20 mmol, 2 eq), chloromethyl ethylcarbonate (2.78 g, 20 mmol, 2 eq) and dry potassium carbonate (2.76g, 20 mmol, 2 eq) in DMF (50 ml) was heated at 60°C for 24 hours (see Supplemental Scheme 1). The residue was treated with ethyl acetate (50 ml) and purified by flash chromatography using a gradient of ethyl acetate/hexane to give pure **ELQ-453** (3.38 g, 86 %) as a white solid. ^1^H-NMR (400 MHz; CDCl_3_): δ 7.49 (ddd, *J* = 9.7, 2.5, 1.4 Hz, 1H), 6.99 (ddd, *J* = 12.2, 8.9, 2.5 Hz, 1H), 5.78 (d, *J* = 1.9 Hz, 2H), 4.25 (q, *J* = 7.1 Hz, 2H), 2.82-2.78 (m, 2H), 2.73 (s, 3H), 1.59-1.28 (m, 13H), 0.91 (t, *J* = 6.9 Hz, 3H). ^13^C-NMR (101 MHz; CDCl_3_): δ 162.79, 162.64, 160.31, 160.17, 158.35, 158.21, 155.88, 155.78, 155.64, 154.2, 149.53, 149.50, 149.39, 149.36, 127.62, 127.59, 109.79, 109.77, 109.70, 109.68, 109.45, 109.40, 109.24, 109.19, 102.9, 102.67, 102.65, 102.4, 92.86, 92.74, 64.6, 31.8, 30.0, 29.4, 29.0, 26.6, 23.7, 22.6, 14.16, 14.07. ^19^F-NMR (376 MHz; CDCl_3_): δ -109.2 (q, J_H_ = 8.8 Hz, 1F), -113.1 (t, J_H_ = 10.4 Hz). HRMS (ESI) m/z for [C_21_H_27_F_2_NO_4_]: Calculated [M+H]^+^: 396.1981, Found [M+H]^+^: 396.1976.

*((5,7-Difluoro-2-methyl-3-undecylquinolin-4-yl)oxy)methyl ethyl carbonate (ELQ-456):* Following the general Procedure B using **ELQ-437** (3.49 g, 10.0 mmol, 1eq), tetrabutylammonium iodide (7.38 g, 20.0 mmol, 2 eq), chloromethyl ethylcarbonate (2.78 g, 20.0 mmol, 2 eq) and dry potassium carbonate (2.76 g, 20.0 mmol, 2 eq) in DMF ( 150 ml) was heated at 60°C for 18 hours (see Supplemental Scheme 1). The residue was treated with ethyl acetate (100 ml) and purified by flash chromatography using a gradient of ethyl acetate/hexane to give pure **ELQ-456** (4.0 g, 89 %) as a white solid. ^1^H-NMR (400 MHz; CDCl_3_): δ 7.46 (ddd, *J* = 9.7, 2.5, 1.4 Hz, 1H), 6.97 (ddd, *J* = 12.2, 8.9, 2.5 Hz, 1H), 5.75 (d, *J* = 1.9 Hz, 2H), 4.23 (q, *J* = 7.1 Hz, 2H), 2.79-2.75 (m, 2H), 2.71 (s, 3H), 1.58-1.50 (m, 2H), 1.42-1.23 (m, 19H), 0.88 (t, *J* = 6.9 Hz, 3H). ^13^C-NMR (101 MHz; CDCl_3_): δ 162.80, 162.66, 160.32, 160.18, 158.36, 158.22, 155.89, 155.86, 155.80, 155.65, 154.2, 149.54, 149.52, 149.40, 149.38, 127.65, 127.63, 127.61, 109.81, 109.79, 109.72, 109.70, 109.47, 109.42, 109.26, 109.21, 103.0, 102.69, 102.67, 102.4, 92.87, 92.75, 64.6, 31.9, 30.0, 29.65, 29.62, 29.43, 29.39, 29.33, 26.6, 23.7, 22.7, 14.18, 14.11. ^19^F-NMR (376 MHz; CDCl_3_): δ -109.2 (q, J_H_ = 8.9 Hz, 1F), -113.1 (t, J_H_ = 10.3 Hz, 1F). HRMS (ESI) m/z for [C_25_H_35_F_2_NO_4_]: Calculated [M+H]^+^: 452.2607, Found [M+H]^+^: 452.2610.

*Ethyl (((6-fluoro-3-heptyl-7-methoxy-2-methylquinolin-4-yl)oxy)methyl) carbonate (ELQ-465).* Following the general Procedure B using **ELQ-433** (1.22 g, 4.0 mmol, 1eq), tetrabutylammonium iodide (2.95 g, 8.0 mmol, 2 eq), chloromethyl ethylcarbonate (1.11 g, 8.0 mmol, 2 eq) and dry potassium carbonate (1.10 g, 8.0 mmol, 2 eq) in DMF (50 ml) was heated at 60°C for 24 hours (see Supplemental Scheme 1). The residue was treated with ethyl acetate (50 ml) and purified by flash chromatography using a gradient of ethyl acetate/hexane 5/5. Second purification by flash chromatography using a gradient of ethyl acetate/hexane to give pure **ELQ-465** (1.0 g, 61 %) as a white solid. ^1^H-NMR (400 MHz; CDCl_3_): δ 7.54 (d, *J* = 11.9 Hz, 1H), 7.41 (d, *J* = 7.8 Hz, 1H), 5.75 (s, 2H), 4.23 (q, *J* = 7.1 Hz, 2H), 4.00 (s, 3H), 2.76 (t, *J* = 7.8 Hz, 2H), 2.70 (s, 3H), 1.58-1.25 (m, 13H), 0.89 (t, *J* = 5.9 Hz, 3H). ^13^C-NMR (101 MHz; CDCl_3_): δ 159.81, 159.79, 157.20, 157.15, 154.2, 153.3, 150.8, 150.33, 150.19, 145.8, 125.1, 116.84, 116.76, 109.47, 109.45, 106.3, 106.1, 92.1, 64.7, 56.2, 31.8, 29.9, 29.7, 29.0, 26.8, 23.5, 22.6, 14.11, 14.09. ^19^F-NMR (376 MHz; CDCl_3_): δ -133.4 (dd, J_H_ = 12.0 Hz, 8.2 Hz, 1F). HRMS (ESI) m/z for [C_22_H_30_FNO_5_]: Calculated [M+H]^+^: 408.2181, Found [M+H]^+^: 408.2182.

*((6-Chloro-3-heptyl-7-methoxy-2-methylquinolin-4-yl)oxy)methyl ethyl carbonate (ELQ-613).* Following the general Procedure B using **ELQ-432** (644 mg, 2.0 mmol, 1eq), tetrabutylammonium iodide (1.48 g, 4.0 mmol, 2 eq), chloromethyl ethylcarbonate (556 mg, 4.0 mmol, 2 eq) and dry potassium carbonate (556 mg, 4.0 mmol, 2 eq) in DMF (25 ml) was heated at 60°C for 24 hours (see Supplemental Scheme 1). The residue was treated with ethyl acetate (25 ml) and purified by flash chromatography using a gradient of ethyl acetate/hexane to give pure **ELQ-613** (510 mg, 60%) as a white solid. ^1^H-NMR (400 MHz; CDCl_3_): δ 7.94 (s, 1H), 7.41 (s, 1H), 5.78 (s, 2H), 4.26 (q, *J* = 7.1 Hz, 2H), 4.03 (s, 3H), 2.80-2.76 (m, 2H), 2.73 (s, 3H), 1.61-1.28 (m, 13H), 0.91 (t, *J* = 6.9 Hz, 3H). ^13^C-NMR (101 MHz; CDCl_3_): δ 161.0, 156.8, 155.6, 154.2, 147.8, 125.1, 123.9, 122.4, 117.5, 108.2, 92.2, 64.8, 56.4, 31.8, 29.9, 29.7, 29.0, 26.7, 23.6, 22.6, 14.13, 14.09. HRMS (ESI) m/z for [C_22_H_30_ClNO_5_]: Calculated [M+H]^+^: 424.1885, Found [M+H]^+^: 424.1881.

*((3-Butyl-6-fluoro-7-methoxy-2-methylquinolin-4-yl)oxy)methyl ethyl carbonate (ELQ-614):* Following the general Procedure B using **ELQ-250**  (526 mg, 2.0 mmol, 1eq), tetrabutylammonium iodide (1.48 g, 4.0 mmol, 2 eq), chloromethyl ethylcarbonate (556 mg, 4.0 mmol, 2 eq) and dry potassium carbonate (556 mg, 4.0 mmol, 2 eq) in DMF ( 25 ml) was heated at 60°C for 24 hours (see Supplemental Scheme 1). The residue was treated with ethyl acetate (50 ml) and purified by flash chromatography twice using a gradient of ethyl acetate/hexane to give pure **ELQ-614** (237 mg, 32%) as a white solid. ^1^H-NMR (400 MHz; CDCl_3_): δ 7.54 (d, *J* = 11.8 Hz, 1H), 7.41 (d, *J* = 8.1 Hz, 1H), 5.75 (s, 2H), 4.23 (q, *J* = 7.1 Hz, 2H), 4.00 (s, 3H), 2.79-2.75 (m, 2H), 2.70 (s, 3H), 1.57-1.51 (m, 2H), 1.47-1.41 (m, 2H), 1.31 (t, *J* = 7.1 Hz, 3H), 0.97 (t, *J* = 7.3 Hz, 3H). ^13^C-NMR (101 MHz; CDCl_3_): δ 159.81, 159.79, 157.21, 157.16, 154.2, 153.3, 150.8, 150.34, 150.20, 145.8, 125.1, 116.81, 116.73, 109.49, 109.47, 106.2, 106.0, 92.1, 64.8, 56.2, 31.8, 26.4, 23.5, 23.0, 14.1, 13.8. ^19^F-NMR (376 MHz; CDCl_3_): δ -133.3 (dd, J_H_ = 11.7 Hz, 8.13 Hz, 1F). HRMS (ESI) m/z for [C_19_H_24_FNO_5_]: Calculated [M+H]^+^: 366.1711, Found [M+H]^+^: 366.1706.

*((3-Butyl-6-chloro-7-methoxy-2-methylquinolin-4-yl)oxy)methyl ethyl carbonate (ELQ-618):* Following the general Procedure B using **ELQ-285**  (560 mg, 2.0 mmol, 1eq), tetrabutylammonium iodide (1.48 g, 4.0 mmol, 2 eq), chloromethyl ethylcarbonate (556 mg, 4.0 mmol, 2 eq) and dry potassium carbonate (556 mg, 4.0 mmol, 2 eq) in DMF ( 25 ml) was heated at 60°C for 24 hours (see Supplemental Scheme 1). The residue was treated with ethyl acetate (50 ml) and purified by flash chromatography using a gradient of ethyl acetate/hexane to give pure **ELQ-618** (90 mg, 12%) as a white solid. ^1^H-NMR (400 MHz; CDCl_3_): δ 7.92 (s, 1H), 7.39 (s, 1H), 5.76 (s, 2H), 4.24 (q, *J* = 7.1 Hz, 2H), 4.01 (s, 3H), 2.79-2.75 (m, 2H), 2.69 (t, *J* = 1.6 Hz, 3H), 1.57-1.51 (m, 2H), 1.48-1.41 (m, 2H), 1.31 (t, *J* = 7.1 Hz, 3H), 0.97 (t, *J* = 7.3 Hz, 3H). ^13^C-NMR (101 MHz; CDCl_3_): δ 161.0, 156.8, 155.6, 154.2, 147.8, 125.0, 123.9, 122.4, 117.4, 108.2, 92.2, 64.8, 56.4, 31.7, 26.4, 23.6, 23.0, 14.1, 13.8. HRMS (ESI) m/z for [C_19_H_24_ClNO_5_]: Calculated [M+H]^+^: 382.1416, Found [M+H]^+^: 382.1414.

*Supplemental Scheme 2: (a) chloromethyl ethyl carbonate, tetrabutyl ammonium iodide, potassium carbonate and DMF, 60°C, 24 h.*

*6-Chloro-7-methoxy-2-methyl-3-(4-(4-(trifluoromethoxy)phenoxy)phenyl)quinolin-4(1H)-one (ELQ-300).* Synthesized (see Supplemental Scheme 2) using a published procedure^2^.

*((6-Chloro-7-methoxy-2-methyl-3-(4-(4-(trifluoromethoxy)phenoxy)phenyl)quinolin-4-yl)oxy)methyl ethyl carbonate (ELQ-331).* Synthesized (see Supplemental Scheme 2) using a published procedure^3^.

*5,7-Difluoro-2-methyl-3-(4-(4-(trifluoromethoxy)phenoxy)-phenyl)quinolin-*

*4(1H)-one (ELQ-400).* Synthesized (see Supplemental Scheme 2) using a published procedure^4^.

*((5,7-Difluoro-2-methyl-3-(4-(4-(trifluoromethoxy)phenoxy)phenyl)quinolin-4-yl)oxy)methyl ethyl carbonate (ELQ-458):* Following the general Procedure B using **ELQ-400** (10.6 g, 23.7 mmol, 1eq), tetrabutylammonium iodide (17.5 g, 47.4 mmol, 2 eq), chloromethyl ethylcarbonate (6.5 g, 47.4 mmol, 2 eq) and dry potassium carbonate (6.5 g, 47.4 mmol, 2 eq) in DMF ( 200 ml) was heated at 60 °C for 24 hours (see Supplemental Scheme 2). The residue was treated with ethyl acetate (250 ml) and purified by flash chromatography using a gradient of ethyl acetate/hexane to give **ELQ-458** (10.74 g). The product was further crystallized in ethyl acetate/hexane to give pure **ELQ-458** (9.31 g) as a white solid. The mother liquor yielded an additional 766 mg for a total **ELQ-458** (10.08 g 77 %). ^1^H-NMR (400 MHz; CDCl_3_): δ 7.53 (ddd, *J* = 9.7, 2.5, 1.4 Hz, 1H), 7.35-7.32 (m, 2H), 7.26-7.23 (m, 2H), 7.13-7.09 (m, 4H), 7.01 (ddd, *J* = 11.8, 9.0, 2.7 Hz, 1H), 5.41 (d, *J* = 1.2 Hz, 2H), 4.08 (q, *J* = 7.1 Hz, 2H), 2.53 (s, 3H), 1.24 (t, *J* = 7.1 Hz, 3H). 13-C NMR (101 MHz; CDCl3): δ 163.53, 163.39, 163.2, 162.0, 161.04, 160.90, 159.29, 159.14, 156.74, 156.69, 156.55, 156.45, 156.44, 156.31, 155.2, 154.0, 150.39, 150.37, 150.25, 150.23, 144.86, 144.84, 144.82, 131.9, 129.7, 127.23, 127.21, 127.19, 122.8, 121.8, 120.1, 119.2, 118.8, 110.14, 110.12, 110.05, 110.03, 109.47, 109.42, 109.27, 109.22, 103.4, 103.11, 103.08, 102.8, 91.77, 91.70, 64.4, 25.1, 14.0. 19-F NMR (376 MHz; CDCl3): δ -58.3 (s, 3F), -107.5 (q, J_H_ = 8.9Hz, 1F), -110.7 (t, J_H_ = 10.2Hz, 1F). HRMS (ESI) m/z for [C_27_H_20_F_5_NO_6_]: Calculated [M+H]^+^: 550.1284, Found [M+H]^+^: 550.1283.

NMR spectra and HPLC chromatograms of ELQ compounds are shown in Supplementary Table 5.

**References**

1. Winter R, Kelly JX, Smilkstein MJ, Hinrichs D, Koop DR, Riscoe MK. Optimization of endochin-like quinolones for antimalarial activity. *Exp Parasitol*. 2011;127(2):545-551. doi:10.1016/j.exppara.2010.10.016

2. Nilsen A, LaCrue AN, White KL, et al. Quinolone-3-Diarylethers: A New Class of Antimalarial Drug. *Science Translational Medicine*. 2013;5(177):177ra37-177ra37. doi:10.1126/scitranslmed.3005029

3. Frueh L, LI Y, Mather MW, et al. Alkoxycarbonate Ester Prodrugs of Preclinical Drug Candidate ELQ-300 for Prophylaxis and Treatment of Malaria. *ACS Infect Dis*. 2017;3(10):728-735. doi:10.1021/acsinfecdis.7b00062

4. Stickles AM, Ting LM, Morrisey JM, et al. Inhibition of Cytochrome bc1 as a Strategy for Single-Dose, Multi-Stage Antimalarial Therapy. *Am J Trop Med Hyg*. 2015;92(6):1195-1201. doi:10.4269/ajtmh.14-0553

**Supplementary Table legends**

**Supplementary Table 1:** Topical application screening compounds. This table lists all the compounds screened by topical application (the first 50 shown in Fig. 1b). Known targets/resistance mechanisms, odds ratio and 95% confidence interval of infection prevalence relative to control (Baptista-Pike method), and where available, SMILES, are listed.

**Supplementary Table 2:** QSAR dataset and descriptors. Tabs are labeled and contain the compounds used for model training and validation, their molecular operating environment (MOE) descriptors, their topical application activity and transformed activities values used for modeling, the models generated by MOE, and the validation parameters for each generated model.

**Supplementary Table 3:** MRM Transitions. This table contains the multiple reaction monitoring (MRM) quantifier and qualifier transitions for each of the ELQs evaluated by mass spectrometry in Fig. 3e.

**Supplementary Table 4:** CACTI Analysis. This table contains the Chemical Analysis and Clustering for Target Identification (CACTI) in silico analysis performed on the topical application library to identify and collate alternate compound names, analogs, relevant citations, possible additional targets.

**Supplementary Table 5:** NMR Spectra and HPLC chromatograms of novel ELQs.
