# Supplementary material for: In vivo screen of Plasmodium targets for mosquito-based malaria control
Source: Nature. 2025 May 21;643(8072):785–93. doi: 10.1038/s41586-025-09039-2 (PMC12267055; doi:10.1038/s41586-025-09039-2)
Supplement: Supplementary file 2 — Reporting Summary [file 41586_2025_9039_MOESM2_ESM.pdf]

Corresponding author(s): Flaminia Catteruccia and Dyann Wirth

Last updated by author(s): Mar 28, 2025

Reporting Summary

Nature Portfolio wishes to improve the reproducibility of the work that we publish. This form provides structure for consistency and transparency in reporting. For further information on Nature Portfolio policies, see our Editorial Policies and the Editorial Policy Checklist.

Statistics

For all statistical analyses, confirm that the following items are present in the figure legend, table legend, main text, or Methods section.

n/a	Confirmed	
☐	☒	The exact sample size (*n*) for each experimental group/condition, given as a discrete number and unit of measurement
☐	☒	A statement on whether measurements were taken from distinct samples or whether the same sample was measured repeatedly
☐	☒	The statistical test(s) used AND whether they are one- or two-sided<br>*Only common tests should be described solely by name; describe more complex techniques in the Methods section.*
☒	☐	A description of all covariates tested
☐	☒	A description of any assumptions or corrections, such as tests of normality and adjustment for multiple comparisons
☐	☒	A full description of the statistical parameters including central tendency (e.g. means) or other basic estimates (e.g. regression coefficient) AND variation (e.g. standard deviation) or associated estimates of uncertainty (e.g. confidence intervals)
☐	☒	For null hypothesis testing, the test statistic (e.g. *F*, *t*, *r*) with confidence intervals, effect sizes, degrees of freedom and *P* value noted<br>*Give P values as exact values whenever suitable.*
☒	☐	For Bayesian analysis, information on the choice of priors and Markov chain Monte Carlo settings
☒	☐	For hierarchical and complex designs, identification of the appropriate level for tests and full reporting of outcomes
☒	☐	Estimates of effect sizes (e.g. Cohen's *d*, Pearson's *r*), indicating how they were calculated

Our web collection on statistics for biologists contains articles on many of the points above.

Software and code

Policy information about availability of computer code

Data collection	No software was used for data collection
Data analysis	GraphPad Prism 10.0; JMP Pro 17; ImageJ version 2.0.0-rc-69/1.52p; SciEX OS version 3.3.1; GATK HaplotypeCaller, GATK version 3.5; GATK CNV pipeline version 4.0; SnpEff version 4.3; 3D7 reference genome from PlasmoDB version 13.0, CACTI tool version 1 (Godinez-Macias & Winzeler, Journal of Chemoinformatics, 2024 doi: 10.1186/s13321-024-00885-2, GitHub: https://github.com/winzeler-lab/CACTI); Python version 3.5; ChemDraw version 22.2; Molecular Operating Environment (MOE) software version 2022.02 (Chemical Computing Group)

For manuscripts utilizing custom algorithms or software that are central to the research but not yet described in published literature, software must be made available to editors and reviewers. We strongly encourage code deposition in a community repository (e.g. GitHub). See the Nature Portfolio guidelines for submitting code & software for further information.

Data

Policy information about availability of data

All manuscripts must include a data availability statement. This statement should provide the following information, where applicable:

- Accession codes, unique identifiers, or web links for publicly available datasets
- A description of any restrictions on data availability
- For clinical datasets or third party data, please ensure that the statement adheres to our policy

Whole genome sequencing data have been deposited in the NCBI Sequence Read Archive (BioProject accession: PRJNA1121409). P. falciparum 3D7 reference

genome (PlasmoDB v13.0) is available at BioProject accession: PRJNA13173 (v13 FASTA: https://plasmodb.org/common/downloads/release-13.0/Pfalciparum3D7/fasta/data/PlasmoDB-13.0_Pfalciparum3D7_Genome.fasta). All other data supporting the findings of this study are available within the article, its Supplementary Information, and source data available from the Harvard Dataverse repository: https://doi.org/10.7910/DVN/UFRFAG.

Research involving human participants, their data, or biological material

Policy information about studies with human participants or human data. See also policy information about sex, gender (identity/presentation), and sexual orientation and race, ethnicity and racism.

Reporting on sex and gender	Human red blood cells (RBCs) and serum were used for the culture of Plasmodium falciparum. Both were sourced from anonymous healthy male donors.
Reporting on race, ethnicity, or other socially relevant groupings	RBCs and serum for P. falciparum culturing was sourced from anonymous healthy O+ white male donors from Research Blood Components (Watertown, Massachusetts, USA) and Interstate Blood Bank (Memphis, Tennessee, USA).
Population characteristics	All additional donor population characteristics were anonymized prior to receiving RBCs or serum and are unknown to the researchers.
Recruitment	No specific recruitment was conducted. Anonymous donors gave blood or serum at the above locations.
Ethics oversight	All experiments were conducted following relevant NIH guidelines and regulations. Samples were sourced by outside vendors (Research Blood Components and Interstate Blood Bank) in accordance with relevant informed consent policies.

Note that full information on the approval of the study protocol must also be provided in the manuscript.

Field-specific reporting

Please select the one below that is the best fit for your research. If you are not sure, read the appropriate sections before making your selection.

☒ Life sciences ☐ Behavioural & social sciences ☐ Ecological, evolutionary & environmental sciences

For a reference copy of the document with all sections, see nature.com/documents/nr-reporting-summary-flat.pdf

Life sciences study design

All studies must disclose on these points even when the disclosure is negative.

Sample size	For infection experiments, researchers aimed for a minimum of >20 mosquitoes per group, per replicate based on previously calculated power analyses (PMID: 30814727, Paton et al. 2019, Nature). In rare instances, fewer mosquitoes were dissected due to poor mosquito feeding and/or mortality. For immunofluoresence assays, 200 parasites or all parasite in the sample (whichever occurred first) were counted per replicate to ensure coverage of a representative sample of parasite midgut forms. For LC-MS experiments, five mosquitoes mosquito midguts were collected per technical replicate to ensure ELQ concentrations were detectable and two technical replicates (of five midguts each) per timepoint were collected to account for possible variability in mosquito midgut size, midgut dissections and handling, and instrument variability. For asexual blood stage dose response curves, three technically replicate wells were used for each compound concentration to account for possible well-to-well variability.
Data exclusions	Infection experiments were excluded if bloodfed control mosquitoes had less than the predetermined 50% oocyst prevalence at 7 dpi as this 1) indicated that this was an atypically poor infection and 2) reduced the experimental power to detect differences in prevalence between control and experimental groups. One technical replicate from one timepoint of the ELQ-458 LC/MS was excluded as it was >4 standard deviations from the mean detection of this sample across two bioreps and two technical replicates and likely represented a detection error.
Replication	The complete topical application screen of 81 compounds was performed once. Any compounds that showed a preliminary reduction in oocyst prevalence were tested at minimum once more for two biological replicates. Four biological replicates were performed for ookinete immunofluoresence assays. The secondary tarsal contact screen of hit topical compounds was performed once. Any compounds that showed a preliminary reduction in oocyst prevalence were tested at minimum once more for two biological replicates.Two biological replicates were performed for ELQ topical and tarsal assays. Two biological replicates with two technical replicates each were performed for ELQ LC/MS. Two to six biological replicates were performed for tarsal contact dose response curves (ELQ-453: six, ELQ-613: four, ELQ combination: three, atovaquone: two). Two to three biological replicates were performed for LDPE and HDPE thin film tarsal contact assays. Two biological replicates were performed for net dipping tarsal contact assays. Three biological replicates with three technical replicates each were performed for asexual blood stage dose response curves. Five replicates were performed for V259L mutant transmission oocysts and three replicates for V259L sporozoites. All attempts at replication were successful.
Randomization	Mosquitoes were collected as pupae and randomly aspirated into cages for experimentation. For oocyst and sporozoite quantification, either all mosquitoes (if the terminal time point) or a random sample of mosquitoes were collected from the cage for dissection. Asexual blood stage P. falciparum experiments were not randomized as different lines were used (WT vs. Cyt B mutants).
Blinding	For the topical application screen, compounds were assigned a random internal identifier to blind researchers to compound identity for the from the point of exposure until analysis. After analysis compound identities were unblinded. Researchers were blinded for 22 hour post-infection immunofluorecense assays, due to potential for human variability in scoring parasite life cycle stages at this time point. After topical applications, each group was anonymized by another member of the laboratory and researchers were subsequently blinded for the remainder of the infection, microscopy, life cycle stage quantification, and data analysis. Two researchers independently counted parasites and scored

them as zygotes, retorts, or ookinetes for each group. Researchers were not blinded for the remaining experiments which entailed quantitative readouts (such as oocyst detection, LC/MS quantification, and asexual blood stage growth). Midgut images were also retained from all infection experiments for analysis verification.

Reporting for specific materials, systems and methods

We require information from authors about some types of materials, experimental systems and methods used in many studies. Here, indicate whether each material, system or method listed is relevant to your study. If you are not sure if a list item applies to your research, read the appropriate section before selecting a response.

Materials & experimental systems

n/a	Involved in the study
☐	☒ Antibodies
☐	☒ Eukaryotic cell lines
☒	☐ Palaeontology and archaeology
☐	☒ Animals and other organisms
☒	☐ Clinical data
☒	☐ Dual use research of concern
☒	☐ Plants

Methods

n/a	Involved in the study
☒	☐ ChIP-seq
☒	☐ Flow cytometry
☒	☐ MRI-based neuroimaging

Antibodies

Antibodies used

Anti-Plasmodium falciparum 25 kDa Gamete Surface Protein (Pfs25), BEI Resources NIAID, NIH: Monoclonal Antibody, Cat. # MRA-28, clone 4B7.

Validation

Pfs25: Monoclonal antibody prepared against the 25-kDa gamete surface protein of Plasmodium falciparum (P. falciparum) (Pfs25) was produced from hybridoma clone 4B7 supernatant. The 4B7 hybridoma cell line (BEI Resources MRA-315) was produced by fusion of mouse myeloma cells with splenocytes from BALB/c mice immunized with recombinant vaccinia virus expressing Pfs25, and boosted with whole P. falciparum gametes. The 4B7 monoclonal antibody binds preferentially to surface protein Pfs25 of P. falciparum.
Citation: Barr, P. J, et al. "Recombinant Pfs25 Protein of Plasmodium falciparum Elicits Malaria Transmission Blocking Immunity in Experimental Animals." J. Exp. Med. 174 (1991): 1203-1208. PubMed: 1940798.

Eukaryotic cell lines

Policy information about cell lines and Sex and Gender in Research

Cell line source(s)

Plasmodium falciparum NF54: BEI Resources (MRA-1000), provided by Carolina Barillas-Mury at the NIH via a BSL-II MTA from BEI Resources.
Plasmodium falciparum TM90C2B: Provided by Michael K. Riscoe at Oregon Health and Science University
Plasmodium falciparum Dd2 I22L: Provided by Michael K. Riscoe at Oregon Health and Science University

Authentication

NF54: WT and mutants verified by whole genome sequencing
TM90C2B: Clinical isolate resistant to atovaquone, first obtained in atovaquone Phase 2 clinical trial in Thailand. PMID: 8651372
Dd2 I22L: ELQ-300 resistant strain generated in Dd2 via ELQ-300 selection. Mutation confirmed by Sanger sequencing. PMID: 25605352

Mycoplasma contamination

All cell lines were routinely test for Mycoplasma contamination and confirmed Mycoplasma free with the exception of the TM90C2B line which was positive for Mycoplasma contamination.

Commonly misidentified lines
(See ICLAC register)

No commonly misidentified cell lines were used in this study.

Animals and other research organisms

Policy information about studies involving animals; ARRIVE guidelines recommended for reporting animal research, and Sex and Gender in Research

Laboratory animals

Anopheles gambiae, G3 strain or RES strain (PMID 37339122), female mosquitoes aged 4-7 days at onset of experiment

Wild animals

No wild animals were used in this study.

Reporting on sex

Female mosquitoes were exclusively used for all experiments in this study, as only female mosquitoes take a blood meal and contribute to the transmission of malaria.

Field-collected samples

No field collected samples were used in the study.

Ethics oversight

Lower invertebrate such as mosquitoes are exempt from relevant regulations regarding the use of animals in research.

Note that full information on the approval of the study protocol must also be provided in the manuscript.

Plants

Seed stocks

Report on the source of all seed stocks or other plant material used. If applicable, state the seed stock centre and catalogue number. If plant specimens were collected from the field, describe the collection location, date and sampling procedures.

Novel plant genotypes

Describe the methods by which all novel plant genotypes were produced. This includes those generated by transgenic approaches, gene editing, chemical/radiation-based mutagenesis and hybridization. For transgenic lines, describe the transformation method, the number of independent lines analyzed and the generation upon which experiments were performed. For gene-edited lines, describe the editor used, the endogenous sequence targeted for editing, the targeting guide RNA sequence (if applicable) and how the editor was applied.

Authentication

Describe any authentication procedures for each seed stock used or novel genotype generated. Describe any experiments used to assess the effect of a mutation and, where applicable, how potential secondary effects (e.g. second site T-DNA insertions, mosiacism, off-target gene editing) were examined.
